# Metabolic Regulation of Sugar Assimilation for Lipid Production in *Aspergillus oryzae* BCC7051 through Comparative Transcriptome Perspective

**DOI:** 10.3390/biology10090885

**Published:** 2021-09-08

**Authors:** Tayvich Vorapreeda, Bhimabol Khongto, Chinae Thammarongtham, Tanawut Srisuk, Kobkul Laoteng

**Affiliations:** 1 Biochemical Engineering and Systems Biology Research Group, National Center for Genetic Engineering and Biotechnology (BIOTEC), National Science and Technology Development Agency (NSTDA), at King Mongkut’s University of Technology Thonburi, Bangkok 10150, Thailand; tayvich.vor@biotec.or.th (T.V.); chinae@biotec.or.th (C.T.); 2 Functional Ingredients and Food Innovation Research Group, National Center for Genetic Engineering and Biotechnology (BIOTEC), National Science and Technology Development Agency (NSTDA), Thailand Science Park, Pathum Thani 12120, Thailand; bhimabol.kho@biotec.or.th; 3 Pilot Plant Development and Training Institute, King Mongkut’s University of Technology Thonburi, Bangkok 10150, Thailand; tanawut.sri@mail.kmutt.ac.th

**Keywords:** *Aspergillus oryzae*, carbon source, lipid production, oleaginous fungi, transcriptome

## Abstract

**Simple Summary:**

Oleaginous fungi are a promising candidate to produce microbial lipids as alternative sources for industrial applications. As lipids are intracellular metabolites with dynamic traits, the fungal ability in utilizing carbon sources for biomass and lipid production is significant in terms of production yield and economic feasibility. This study aimed to explore the metabolic regulation in lipogenesis of oleaginous *Aspergillus oryzae* BCC7051 at the transcriptional level. Through comparative transcriptome analysis, a set of differentially expressed genes (DEGs) between the xylose- and glucose-grown cultures (C5 and C6 cultures) at fast-growing and lipid-accumulating stages were identified and functionally categorized into transporter proteins and cellular processes. Combining with the growth and lipid phenotypes, the transcriptome results pointed to a crucial link between sugar assimilation, energy, lipid, and other metabolisms in *A. oryzae* for leveraging the metabolic flux from xylose to fatty acid and lipid biosynthesis in render the oleaginous features. This study provides a remarkable insight in guiding strain optimization and bioprocess development using renewable feedstocks from agroindustrial residues.

**Abstract:**

Microbial lipid production with cost effectiveness is a prerequisite for the oleochemical sector. In this work, genome-wide transcriptional responses on the utilization of xylose and glucose in oleaginous *Aspergillus oryzae* were studied with relation to growth and lipid phenotypic traits. Comparative analysis of the active growth (t1) and lipid-accumulating (t2) stages showed that the C5 cultures efficiently consumed carbon sources for biomass and lipid production comparable to the C6 cultures. By pairwise comparison, 599 and 917 differentially expressed genes (DEGs) were identified in the t1 and t2 groups, respectively, in which the consensus DEGs were categorized into polysaccharide-degrading enzymes, membrane transports, and cellular processes. A discrimination in transcriptional responses of DEGs set was also found in various metabolic genes, mostly in carbohydrate, amino acid, lipid, cofactors, and vitamin metabolisms. Although central carbohydrate metabolism was shared among the C5 and C6 cultures, the metabolic functions in acetyl-CoA and NADPH generation, and biosynthesis of terpenoid backbone, fatty acid, sterol, and amino acids were allocated for leveraging biomass and lipid production through at least transcriptional control. This study revealed robust metabolic networks in the oleaginicity of *A. oryzae* governing glucose/xylose flux toward lipid biosynthesis that provides meaningful hints for further process developments of microbial lipid production using cellulosic sugar feedstocks.

## 1. Introduction

Fungal Biotechnology has contributed to the global challenges for economic and enviromental benefits based on the strategic models of bioeconomy and circular economy. Biotransformation of diverse organic and inorganic feedstocks to valuable metabolites is a plausible process of fungal systems, which offers substantial potential for the development of biotechnological production. Filamentous fungi play a significant role in biomanufacturing primary and secondary metabolites for diverse industrial applications [1]. Of them, *Aspergillus oryzae* is an industrially important strain with a generally recognized as safe (GRAS) status. It is a nonaflatoxin producing strain rendering superior growth performance over other microbial platforms in terms of cell robustness to surrounding environments and existing substrates [2,3]. The accumulative genome datasets of *A. oryzae* strains (https://www.ncbi.nlm.nih.gov/genome/?term=Aspergillus; accessed on 2 April 2021) have permitted significant progress in both fundamental and applied research [4,5,6,7,8]. Apart from the enzyme, amino acid, and organic acid production, *A. oryzae* has also been recognized as an oleaginous strain with capability in lipid production [9]. Several studies demonstrated that *A. oryzae* has a potential as a cell chassis for production of *n*-6 polyunsaturated fatty acids (PUFAs) by engineering fatty acid and lipid biosynthetic pathways, thus yielding γ-linolenic and dihomo-γ-linolenic acids [8,10,11]. 

To expand on the sustainable production of specialty and commodity lipids by the fungal platform, the regulatory mechanisms underlying lipogenesis have been studied. Genome characterization of a selected oleaginous strain of *A. oryzae* BCC7051 revealed the existence of multiple enzyme isoforms involved in the fatty acid synthesis. It also shared metabolic features among other oleaginous yeasts and filamentous fungi [3], which coincide with the comparative genomic analysis describing the relationship between carbohydrate, lipid, and amino acid metabolisms in the acetyl-CoA generation for lipid overproduction [12]. A comparative kinetic modeling of cell growth and lipid production in the BCC7051 strain could distinguish three lipid-producing stages on glucose cultivation, including low lipid-producing, lipid accumulation, and lipid turnover stages [13]. Although glucose is a preferred carbon source for microbial lipid production, xylose and cellulosic sugars derived from nonfood biomass could be efficiently used for lipid production by the oleaginous fungus *Mortierella isabellina* [14,15]. In context of the circular bioeconomy, low-cost feedstocks and agroindustrial by-products have been tested for production of various microbial lipid products ranging from biofuels to high-value functional lipids with prospective applications in energy, feed, food, pharmaceultical, nutraceutical, and olechemical sectors [16,17,18]. Nevertheless, the cellular mechanism in channeling xylose or cellulosic sugars to lipid production of the oleaginous fungi remain largely unexplored. Based on the ability of sugar utilization in *A. oryzae* BCC7051, in this work, we investigated the metabolic regulation of lipid production through comparative transcriptome and phenotypic analysis. The differentially expressed genes (DEGs) between the cultures grown on xylose and glucose at different growth stages were identified and functionally categorized into the relevant metabolic pathways to capture more comprehensive interactive networks of cellular metabolisms, which contribute to the carbon uptake and utilization and lipid production of the oleaginous *A. oryzae* BCC7051.

## 2. Materials and Methods

### 2.1. Fungal Strain and Cultivation Conditions

The wild type *A. oryzae* strain BCC7051 (haploid strain), which was obtained from the BIOTEC Culture Collection (BCC), Thailand, was used throughout this work.

For inoculum preparation, the solid-state fermentation of *A. oryzae* BCC7051 was carried out using polished rice grain as a substrate. We added 20 mL of distilled water into 20 g of polished rice grain in a 250 mL Erlenmeyer flask and then sterilized it at 121 °C for 15 min. Subsequently, the sterilized rice grain was inoculated with 200 µL of spore suspension (1 × 10^8^ spores mL^−1^) and incubated at 30 °C for 5 days as previously described [19]. The fungal spores were then harvested by suspension with 0.05% (*v*/*v*) Tween-80 solution, filtered through a sterile Miracloth (Merck, Darmstadt, Germany), and counted on a hemocytometer.

For submerged fermentation, *A. oryzae* BCC7051 was cultivated in a 5 L bioreactor (Biostat B-DCU, Sartorius, Goettingen, Germany) using a semisynthetic medium (SM), by which one litre consisted of 5.0 g yeast extract, 8.2 g KH_2_PO_4_, 0.5 g MgSO_4_·7H_2_O, 0.1 g CaCl_2_·7H_2_O, 0.2 g NH_4_Cl, 10 mg MnSO_4_·H_2_O, 0.5 mg CuSO_4_·5H_2_O, 15 mg FeCl_3_·6H_2_O, 7.5 mg ZnSO_4_·7H_2_O, and 80 g of carbon source. Two carbon sources, xylose and glucose, were variables for *A. oryzae* cultivations. The spore suspension was inoculated into 3 L SM to obtain a final concentration of 10^6^ spores mL^−1^. Fungal fermentation was carried out at 30 °C with an agitation rate of 500 rpm, air flow rate of 1.0 vvm, and pH at 4.5. The cultures using xylose (C5 culture) and glucose (C6 culture) were grown at logarithmic and late logarithmic stages, which were termed as active growth (t1) and lipid-accumulating (t2) stages, respectively.

### 2.2. Growth and Residual Sugar Measurements

Mycelial cells were harvested by filtering through Miracloth and then subjected to hot-air drying at 60 °C to obtain a constant weight. The fungal biomass was represented as dry cell weight (DCW). 

Residual xylose or glucose concentrations in the fermented broths were measured using high performance liquid chromatography (HPLC; Dionex Ultimate 3000, Thermo Fisher Scientific Inc., Waltham, MA, USA) equipped with an Aminex^®^ HPX-87H ion exclusion column (Bio-Rad Laboratories, Hercules, CA, USA) and a refractive index detector. The isocratic mode with the mobile phase (18 mM H_2_SO_4_ solution) was operated at a flow rate of 0.6 mL min^−1^ at 60 °C for 30 min. 

### 2.3. Fatty Acid and Lipid Analysis

Dried mycelia were subjected to preparation of fatty acid methyl esters (FAMEs) using the direct transmethylation method [20]. The FAMEs samples were analyzed by gas chromatography (GC; Agilent 7890B, Santa Clara, CA, USA) equipped with a HP-88 capillary column (30 m × 250 μm × 0.2 μm, Agilent, Santa Clara, CA, USA) and a flame ionization detector. The temperatures of the column and detector were set at 140–240 °C and 280 °C, respectively. Helium was used as a carrier gas with a constant flow rate of 1.0 mL min^−1^. Individual fatty acids were identified by comparing their retention times of chromatographic peaks with those of FAMEs standards (Sigma–Aldrich, St. Louis, MO, USA). Fatty acid proportions in total fatty acid were calculated from the respective area of chromatographic peaks using heneicosanoic acid (C21:0) as an internal standard.

The total lipid of dried mycelia was extracted by using the method modified from Folch et al. [21]. Lipid classes were analyzed by HPLC with a charged aerosol detector (CAD) as previously reported [22]. Individual lipid classes, triacylglycerol (TAG), including steryl ester (SE), free fatty acid (FFA), and phospholipids (PL), were identified by comparing their retention times with those of authentic lipid standards (Sigma, USA). Proportion of each lipid class in the total lipid was calculated from the respective chromatographic area.

### 2.4. RNA Extraction and Sequencing

Total RNA was extracted from the C5 and C6 cultures of *A. oryzae* grown at active growth (C5_t1 and C6_t1) and late logarithmic (C5_t2 and C6_t2) phases using the RNeasy mini kit (QIAGEN). RNA quality and concentration were measured using Agilent 2100 bioanalyzer NanoDrop^TM^. Equal amounts of total RNA samples extracted from three independent cultures of each condition were pooled. Then, the cDNA library construction and RNA sequencing were performed using Novagene’s Illunina to yield paired end reads.

### 2.5. RNA Sequence Data Analysis

After RNA sequencing, raw reads were first processed through in-house perl scripts. In this step, clean reads were obtained by removing reads containing adapters, reads containing a high content of unknown base (poly-N), and low-quality reads from the raw reads. In parallel, Q20, Q30, and GC content of the clean data were calculated. The downstream analyses were based on the clean data with high quality. Next, the reference genome of *A. oryzae* BCC7051 (GCA_002007945.1) [3] indexed through Bowtie (v2.2.3) [23] and TopHat (v2.0.12) [24] programs was used for mapping the high-quality paired-end clean reads onto the reference genome with “mismatch 2” as the parameter. HTseq v0.6.1 [25] was applied to count the reads numbers mapped to individual genes. Then, Cufflinks (v2.2.1) [26] was used for estimating gene expression levels, which were normalized based on both gene length and library size as a FPKM (fragments per kilobase of transcript per million mapped reads) value [27]. Prior to detecting DEGs, the read counts for each sample were adjusted using the edgeR program (v3.0.8) [28], and then the differential gene expression analysis between two experimental sets was performed using DEGseq (v1.12.0) [29] with a *p* value of 0.005 and absolute log2 (fold change) of 1, which were both set as thresholds for significantly differentially expression.

### 2.6. Transcriptome Annotation and Pathway Analysis

To obtain comprehensive information of transcriptome data, all transcripts derived from the RNA-seq data were subjected to BLASTN search against the genome sequences of *A. oryzae* strain BCC7051 (GCA_002007945.1) and RIB40 (GCA_000184455.3), whose annotation data have been deposited at NCBI database. In addition, all transcripts were searched against the Kyoto Encyclopedia of Genes and Genomes (KEGG) database [30] using BLASTX for improving its annotation data. The enzyme commission (EC) numbers and the relevant KEGG metabolic pathways were retrieved. The gene ontology (GO) assignment was used to classify the annotated information of all transcripts. The GOseq R package [31] was employed to retrieve GO annotations for describing biological processes, molecular functions, and cellular compartmentation.

## 3. Results

### 3.1. Growth Kinetic and Lipid Production of A. oryzae BCC7051 on Different Sugars

At active growth phase (t1), the growth of the C5 and C6 cultures of *A. oryzae* were comparable, which was indicated by their biomass titers (DCW) and biomass productivities (Q_X_), as shown in Table 1. However, the C5 cultures consumed less carbon source for biomass production as compared with the C6 cultures, which were observed in both active growth and late log phases, as clearly shown by residual sugar concentrations and sugar consumption rates (Q_S_). As a consequence, the biomass yield on sugar (Y_X/S_) in the C5 cultures was significantly higher than that of the C6 cultures, revealing that this fungus efficiently used xylose for biomass production. However, the Q_X_ value of the C5 culture (96 h cultivation) grown at the late growth stage (C5_t2) was lower than that of the C6_t2 culture (72 h cultivation), which was caused by the different cultivation times between these cultures for entering the lipid-accumulating stage. 

It was found that the lipid content in the fungal cells markedly increased at the late logarithmic phase as compared to the active growth phase. This phenomenon was found in both cultures using glucose and xylose. Notably, the sugars remained available in both cultures, highlighting the excess carbon cultivation condition (Table 1, Figure S1), which has been reported to trigger intracellular lipid accumulation at the end of the growth stage (late logarithmic phase) of several oleaginous strains that was caused by the limitation of some nutrients or nutrient imbalance for cell growth [32,33]. Although the lipid content and lipid yield on sugar (YP_P/S_) in the C6_t2 culture were lower than those of the C5_t2 culture, there was no difference in the lipid productivities (Q_P_) among the cultures. Notably, the lipid content in DCW of both culures of *A. oryzae* seemed to be lower than other oleaginous microbes [17,32], indicating that the cultivation conditions might be not optimal for maximizing lipid production. It has been reported that there is discrimination in abiotic and biotic factors attributing the oleaginicity of individual strains [33].

The proportion of TAG and PL, which were neutral and polar lipids, respectively, were not significantly different between the C5_t2 and C6_t2 cultures. The SE proportion of the C5_t2 culture was higher than that of the C6_t2 culture, whereas the FFA proportions of the C5 cultures were less than those of the C6 cultures (Table 2). Fatty acid analysis showed that oleic acid (C18:1Δ^9^) and linoleic acid (C18:2Δ^9,12^) were major components in TFA, which were similarly found in both xylose and glucose cultures. The saturated and monounsaturated fatty acids (C18:0 and C18:1Δ^9^) of the C5 and C6 cultures proportionally increased at late logarithmic phase when compared with those of the active-growing cultures (Table 3), whereas the diene fatty acid (C18:2Δ^9,12^) proportions decreased in the lipid-accumulating stage. This similar fashion in the altered fatty acid composition at different growth phases of *A. oryzae* has been reported for oleaginous Zygomycetes, in which the oleic acid proportion substantially increased during cell growth [34].

### 3.2. Genome-Wide Transcriptome Data of A. oryzae BCC7051 

The DEGs of the C5 and C6 cultures of *A. oryzae* BCC7051 grown at different growth phases (t1 and t2) were investigated by comparative transcriptome analysis. The results showed that total raw reads were obtained from four samples accounting for an average of 44.18 Megabase pairs (Mb). After data cleanup and quality checks, the high-quality reads with a Q30 value in an average of more than 92.8% were obtained. Using Bowtie2 and TopHat [23,24], about 89.5% of the high-quality reads could be mapped onto the reference genome of *A. oryzae* BCC7051 (GCA_002007945.1), as summarized in Table S1. Assembly of the high-quality reads revealed that a total of 13,186 transcripts were generated, and FPKM values of the gene expressions are shown in Figure S2.

### 3.3. Differential Gene Expression and Functional Assignment of the A. oryzae Cultures Using Different Carbon Sources

**Figure 1:**
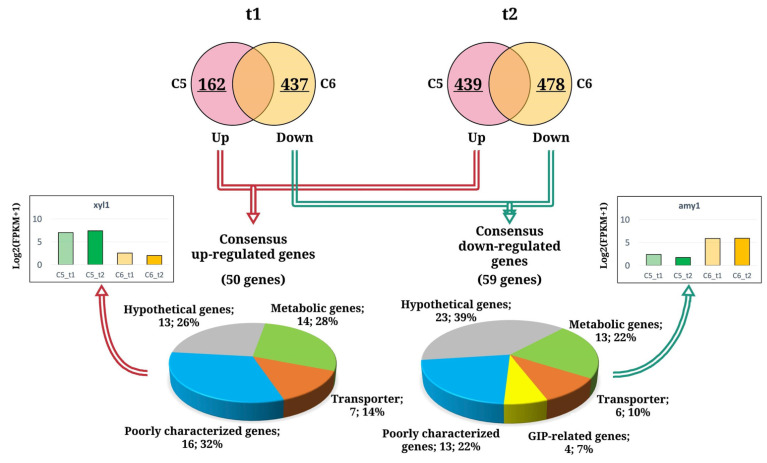
The consensus up- and downregulated DEGs of *A. oryzae* BCC7051 across pairwise comparison between the glucose-grown cultures (C5_t1 and C5_t2) and the xylose-grown cultures (C6_t1 and C6_t2). The functional classification of significantly DEGs was based on KEGG and GO annotation.

To explore the genome-wide expression of *A. oryzae* BCC7051 on different sugars, the transcript levels of the C5 cultures were compared to those of the C6 cultures that were grown at the same growth phases, consisting of group 1 (C5_t1 vs. C6_t1) and group 2 (C5_t2 vs. C6_t2). For group 1, there were 599 DEGs identified by pairwise comparison between the C5_t1 and C6_t1 cultures, which included 162 upregulated genes and 437 downregulated genes. A total of 917 DEGs were identified between the C5_t2 and C6_t2 samples (group 2), by which 439 and 478 genes were up- and downregulated, respectively. By comparing the data set of DEGs between groups 1 and 2, we found that exclusively 50 DEGs with upregulated expression were shared among the C5 cultures grown at different phases as shown in Figure 1. The 59 DEGs with consensus downregulated expression were found in the C6 cultures. In addition, the consensus up- and downregulated DEGs of *A. oryzae* in response to the utilized carbon sources are depicted in Figure 1 and Table S2.

**Figure 2:**
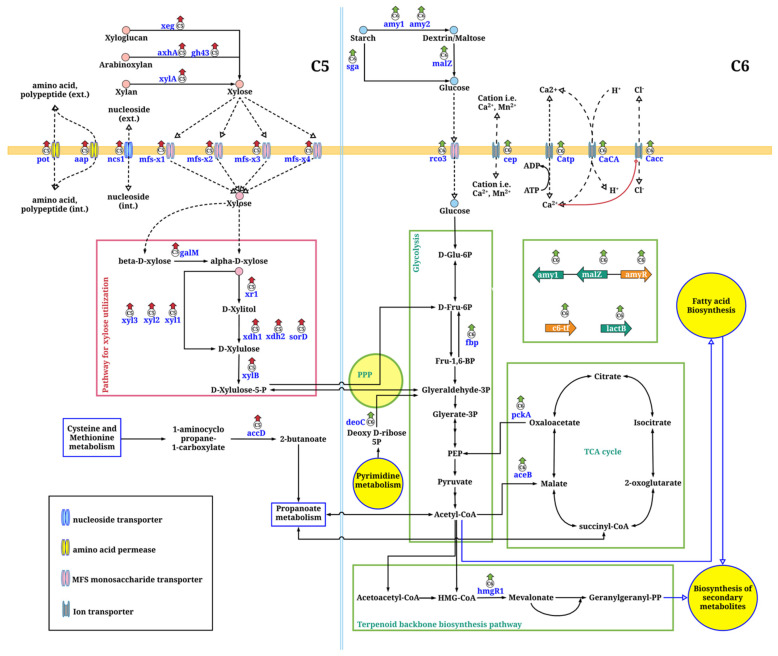
Comparison of metabolic routes of *A. oryzae* BCC7051 between the cultures using C5 (xylose) and C6 (glucose) sugars as carbon sources. At outside of the fungal membrane (top panel), the xylolytic and amylolytic enzymes involved in the consumption of xylose and glucose, respectively, are illustrated. The membrane transport proteins are shown (orange bar). Red- and green-colored arrows above gene symbols indicate significantly upregulated expression of genes at xylose and glucose cultivations, respectively.

The functional analysis using KEGG category showed that majority of the consensus DEGs were hypothetical or poorly characterized genes, which indicated how little is known about the function of these genes in *A. oryzae*. For the annotated DEGs, they were assigned into three main functional categories, including (i) extracellular proteins involved in the polysaccharide-degrading enzyme system, (ii) membrane transport proteins, and (iii) the genes involved in cellular processes as illustrated in Figure 2. The relative expression levels of these genes (FPKM values) are shown in Table S2. The comparative transcriptome analysis indicated that these genes were substantially attributed by sugar types and growth stages as following sections.

### 3.4. Metabolic Network of Xylose Assimilation for A. oryzae Growth through Comparative Transcriptome Analysis

Using xylose as a sole carbon source, four genes of *A. oryzae* BCC7051 encoding polysaccharide-degrading enzymes with xylanolytic activities, which are involved in xylan hydrolysis (xylan beta-D-xylosidase (xylA), and glycoside hydrolase family 43 (gh43)), and the side-chain cleaving enzymes required for the removal of side substituents of heteroxylans (xyloglucan-specific endo-beta-1,4-glucanase (xeg) and arabinofuranosidase (axhA)), were significantly upregulated as compared to those of the C6 cultures. It was also found that seven membrane transporters were transcriptionally upregulated in the C5 cultures (Table S2), in which four of them were annotated as major facilitator superfamily (MFS) transporters (mfs-x1, mfs-x2, mfs-x3 and mfs-x4), which are single polypeptide secondary carriers for the transport of small solutes in response to ion gradients [35]. For the other transporter genes, the expressions of amino acid permease (aap), oligopeptide POT family transporter (pot) and nucleoside transporter (ncs1) in the C5 cultures were also higher than the C6 cultures.

Considering intracellular metabolisms, nine genes were significantly upregulated in the C5 cultures (Figure 2). Among them, seven genes encoded the enzymes in xylose utilization pathway, including three alpha-xylosidases (xyl1, xyl2 and xyl3), two xylulose reductases (xdh1 and xdh2), a xylose reductase (xr1) and a xylulokinase (xylB), which are responsible for converting xylose to xylulose-5-P. In addition, the aldose 1-epimerase (galM) and L-iditol 2-dehydrogenase (sorD) genes, both involved in the interconversion between α-form and β-form sugars [36], and the zinc-dependent interconversion of polyols to their respective ketoses [37], respectively, were upregulated in the C5 cultures. These results revealed that the upregulated expression of a set of genes in the xylose utilization pathway and nonoxidative pentose phosphate pathway (PPP) might associate with serial transformantion and metabolic flow of xylose toward the glycolysis for generating metabolic energy and acetyl-CoA for cell growth and fatty acid biosynthesis, as summarized in Figure 2. Additionally, 1-aminocyclopropane-1-carboxylate deaminase (*accD*) expression was significantly upregulated in the C5 cultures. The accD, which is responsible for breaking down 1-aminocyclopropane-1-carboxylate (ACC) to ammonia and α-ketobutyrate, can be further metabolized for growth through propanoate metabolism and key metabolic pathways, including glycolysis, and the citrate cylce (TCA cycle) [38].

### 3.5. Metabolic Network of Glucose Assimilation for A. oryzae Growth through Comparative Transcriptome Analysis

Using glucose for fungal growth, the expression levels of genes coding for membrane proteins, extracellular proteins, and proteins involved in cellular processes were relatively higher than those of the C5 cultures (Figure 2). Five transporters were transcriptionally induced at different levels, which were a sugar transporter (rco3) and four ion transporters/channels, including cation transporters (cation efflux protein (cep), calcium-transporting ATPase (Catp), calcium/proton antiporter (CaCA)), and anion transporter (calcium-activated chloride channel-domain-containing protein (Cacc)). The sequence analysis showed that the rco3 sequence of *A. oryzae* BCC7051 had a similarity to the high-affinity glucose transporter (RCO3) of *Neurospora crassa* containing 12 membrane-spanning domains, which are signature features of sugar transporters [39]. The previous study also demonstrated that RCO3 of *N. crassa* was required for glucose transport activity and might play a role in sensing glucose [39,40]. Indeed, we postulated that the rco3 protein in *A. oryzae* BCC7051 might be a glucose transporter that also acted as a glucose sensor. It is well known that the uptake of glucose also depends on maintenance of the plasma membrane proton gradient.

The expression levels of the genes encoding amylolytic enzymes in the C6 cultures were relatively higher than those of the C5 cultures, which were alpha-amylase (amy1 and amy2), alpha-glucosidase (malZ), and glucoamylase (sga). In addition, we found that a gene encoding amylase cluster transcriptional regulator (AmyR) was expressed at a high level and also upregulated particularly in the C6 cultures. Integrative analysis with the genome sequence of the strain BCC7051 [3] revealed that the *amyR* gene was located adjacent to the amylolytic enzyme cluster encoding for amy1 and malZ. In carbohydrate metabolism, several key genes were upregulated in the C6 cultures of *A. oryzae*, which were fructose-1,6-bisphosphatase (*fbp*), phosphoenolpyruvate carboxykinase (*pckA*), malate synthase (*aceB*), and deoxyribose-phosphate aldolase (*deoC*) genes, indicating that the metabolic functions in gluconeogenesis and glyoxylate cycle were active during the glucose growth rather than the C5 cultures. It seems that fbp, pckA, aceB, and deoC might be key enzymes for the generation of precursors for growth and fatty acid biosynthesis via PPP and TCA under glucose cultivations of *A. oryzae*.

Three DEGs (*hmgR1*, *hmgR2*, and *hmgR3*), which encoded putative hydroxymethylglutaryl coenzyme A (HMG-CoA) reductases involved in the biosynthesis of terpenoid backbone and sterol, were identified in the C6 cultures. However, only *hmgR1* gene was strongly overexpressed in the lipid-accumulating phase, whereas *hmgR2* and *hmgR3* transcript levels did not significantly alter between different growth phases of C6 cultures (Table S3). The previous study demonstrated that glucose was an attribute for inducing the activity of HMG-CoA reductase in the resting yeast cells, yielding the increase of sterol content [41,42]. The HMG-CoA reductase also tightly participates in the sterol biosynthesis when cultivated in glucose [41,42]. 

### 3.6. Transcriptional Alterations at Lipid Accumulation Phases of A. oryzae 

**Figure 3:**
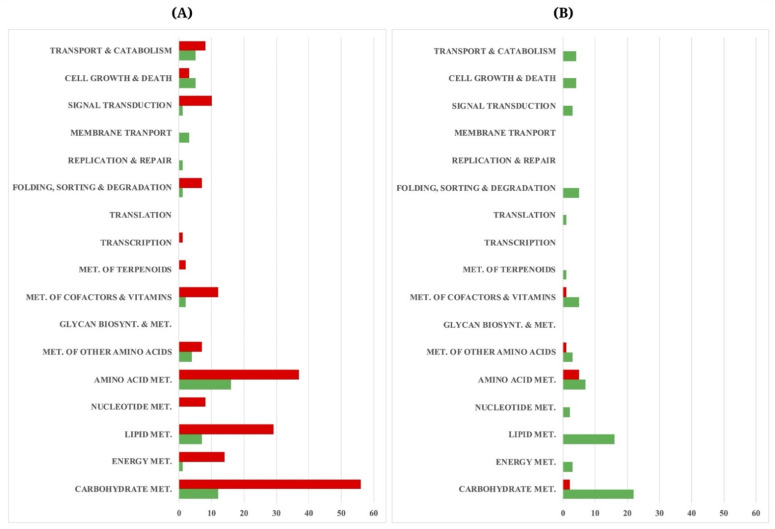
DEGs of the *A. oryzae* cultures at active growth and lipid-accumulating phases. The *x*-axis indicates the number of DEGs, and the *y*-axis represents the metabolic pathways based on KEGG database. (**A**) C5_t1 vs. C5_t2 derived from the analysis between 36- and 96 h cultures using xylose, and (**B**) C6_t1 vs. C6_t2 derived from the analysis between 36- and 72 h cultures using glucose. Red and green bars indicate the up- and downregulated genes, respectively.

The DEGs with significant fold change (at least ≥2-fold, and at a probability level of *p* ≥ 0.005) were identified between diffirent growth phases by mapping read counts of the genes expressed in the C5 and C6 cultures. In the xylose cultivations, we found the altered expressions of 878 genes in the C5_t2 culture. Of them, 455 genes were upregulated (transcript levels increased; t1 < t2), whereas 423 were downregulated in the slow-growth culture using xylose (C5_t2) (transcript levels decreased; t1 > t2). When using glucose, a total of 448 DEGs were identified between the C6_t1 and C6_t2 cultures, of which 167 and 281 were up- and downregulated, respectively. The KEGG pathway enrichment analysis showed that the DEGs of *A. oryzae* involved in metabolic pathways of several metabolisms were significantly attributed by growth stage in addition to the carbon sources, as shown in Figure 3. In particular, the responsive genes were involved in central carbon metabolism (the glycolysis, PPP, and TCA cycle). Moreover, the genes in lipid metabolism (fatty acid and sterol biosynthetic pathways) and amino acid metabolism were also transcriptionally regulated by the variables mentioned (Table S4).

**Figure 4:**
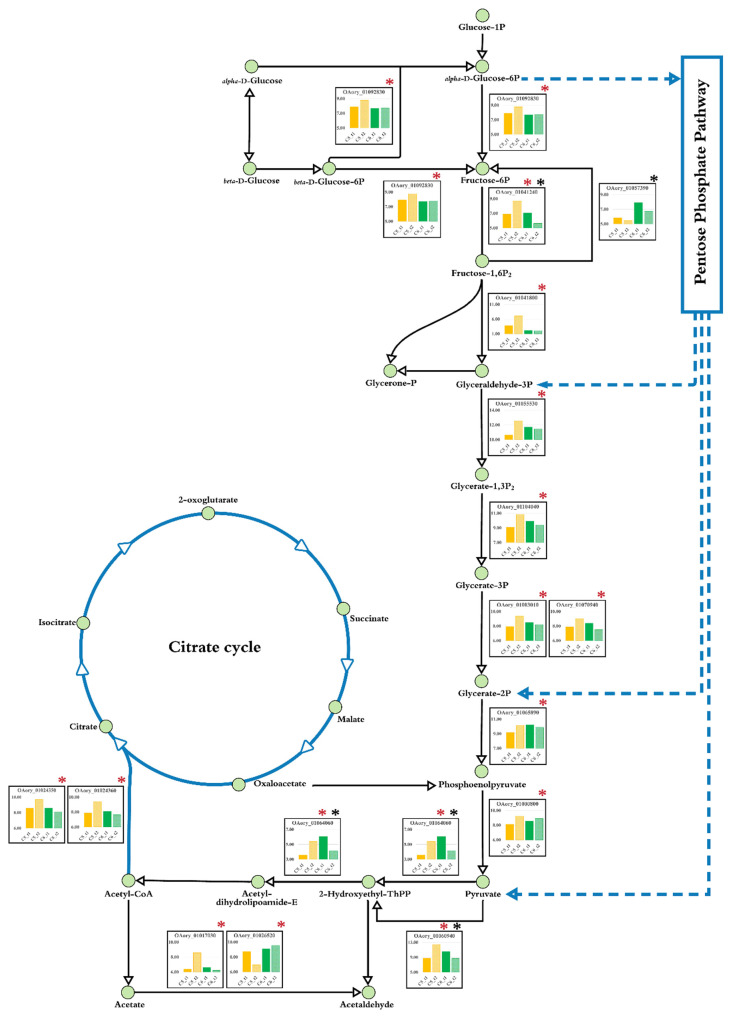
Expression of the genes involved in the carbohydrate metabolic pathway of *A. oryzae* BCC7051. The expression levels of individual genes were transformed to logarithmic space by using using log2(FPKM + 1). The C5_t1 (orange bar) and C5_t2 (light orange bar) represent the xylose-grown cultures at active growth and lipid-accumulating phases, respectively. The C6_t1 (green bar) and C6_t2 (light green bar) indicate the glucose-grown cultures at active growth and lipid-accumulating phases, respectively. Red and black asterisks indicate significant DEGs of the cultures using xylose and glucose, respectively.

Comparing the C5 cultures at different growth stages, at least 10 genes involved in key steps in the glycolysis pathway were transcriptionally upregulated in the lipid-accumulating phase (C5_t2), as shown in Figure 4. These included glucose 6-phosphate isomerase (OAory_01092830), 6-phosphofructokinase (OAory_01041240), fructose-biphosphate aldolase (OAory_01041800), glyceraldehyde 3-phosphate dehydrogenase (OAory_01055530), phosphoglycerate kinase (OAory_01104040), phosphoglycerate mutase (OAory_01003010 and OAory_01070940), enolase (OAory_01065890), pyruvate kinase (OAory_01000800), and pyruvate decarboxylase (OAory_01060940). We also found that the expressions of both subunits of ATP-citrate synthase (OAory_01024350 and OAory_01024360), which catalyze the transformation of ADP, acetyl-CoA and oxaloacetate to ATP, citrate and CoA, respectively, were upregulated in the C5_t2 culture.

**Figure 5:**
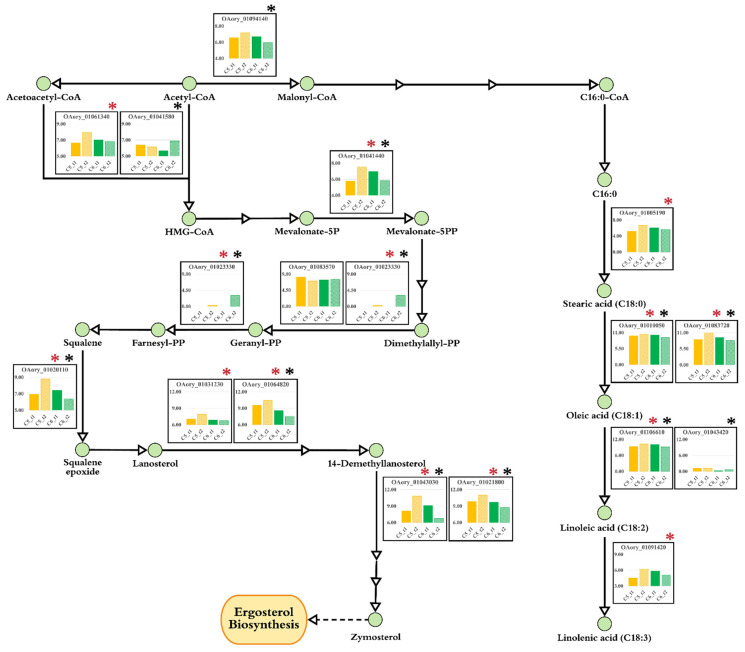
Expression of the genes involved in the fatty acid and sterol biosynthetic pathways of *A. oryzae* BCC7051. The expression levels of individual genes were transformed to logarithmic space using log2(FPKM + 1). The C5_t1 (orange bar) and C5_t2 (light orange bar) represent the xylose-grown cultures at active growth and lipid-accumulating phases, respectively. The C6_t1 (green bar) and C6_t2 (light green bar) indicate the glucose-grown cultures at active growth and lipid-accumulating phases, respectively. Red and black asterisks indicate significant DEGs of the cultures using xylose and glucose, respectively.

Focusing on the ergosterol biosynthesis [43,44,45], we found that three genes encoding hydroxymethylglutaryl-CoA synthase 2 (HMGCS2; OAory_01061340), phosphomevalonate kinase (OAory_01041440) and geranylgeranyl diphosphate synthase (OAory_01023330), which are involved in metabolic reactions of the terpenoid backbone biosynthetic pathway (mevalonate pathway), were upregulated in the C5_t2 culture. As shown in Figure 5, the upregulated expressions of nine genes involved in the sterol biosynthesis pathway (postsqualene pathway) were also found in the C5_t2 culture, including squalene monooxygenease (erg1; OAory_01020110), two genes of sterol 14-alpha-demethylases (OAory_01031230 and OAory_01064820), two genes of delta 14-sterol reductase (erg24; OAory_01028620 and OAory_01050620), C-5 sterol desaturase (erg25; OAory_01014460), sterol 24-C-methyltransferase (erg3; OAory_01027770), and two genes of methylsterol monooxygenases (erg6; OAory_01043030 and OAory_01021800) (Figure 5 and Table S4). 

For fatty acid modification, five metabolic genes were upregulated at late logarithmic phase when using xylose, including stearoyl-CoA desaturases (delta-9; OAory_01010050 and OAory_01083720), fatty acid desaturase (OAory_01091420), oleate desaturase (delta-12; OAory_01106610), and fatty acid elongase (ELO1; OAory_01005190), as shown in Figure 5. In amino acid metabolism, the genes involved in alanine, aspartate, glutamate, and tyrosine metabolisms were markedly upregulated in the C5_t2 culture. These included glutamate synthase (glt; OAory_01081090), glutamate dehydrogenase (gud; OAory_01030390), glutamate decarboxylase (gad; OAory_01046690), adenylosuccinate lyase (adsl; OAory_01034360), primary-amine oxidase (aoc; OAory_01012920 and OAory_01018570), 4-hydroxyphenylpyruvate dioxygenase (hpd; OAory_01064800), and tyrosinase (tyr; OAory_01105320). Moreover, the genes involved in the metabolism of glycine, serine, and threonine were also upregulated, including threonine dehydratase (tdc; OAory_01028840), tryptophan synthase (trp; OAory_01035660), and phosphoglycerate mutase (pgm; OAory_01003010 and OAory_01070940). 

In the glucose cultivations, the expression of genes in the carbohydrate metabolism were downregulated in the lipid-accumulating phase of *A. oryzae* (C6_t2) as compared to the active growth culture (C6_t1). These DEGs included fructose-1,6-bisphosphatase (OAory_01057390), 6-phosphofructokinase (OAory_01041240), and pyruvate decarboxylase (OAory_01060940), which have been shown to be catalytically important for the gluconeogenesis [46], the glycolysis [47], and the synthesis of cytosolic acety-CoA [48], respectively. For lipid metabolism, the expression of acetyl-CoA carboxylase (ACC; OAory_01094140) was slightly downregulated in the C6_t2 culture, whereas there was no significant difference in the ACC expressions between the active growth and lipid-accumulating phases of the C5 cultures. Although ACC cataylzes the first commiting step of *de novo* fatty acid biosynthesis, its activity did not link to lipid accumulation in *A. oryzae*, similar to some oleaginous microbes [16,49,50,51], postulating that the lipid biosynthesis may be controlled by feedback inhibition mechanism. Moreover, the intracellular lipids, mostly neutral lipids, can be also produced by other pathways, i.e., sterol biosynthesis. For fatty acid modification, the stearoyl-CoA desaturase (delta-9; OAory_01010050 and OAory_01083720) and oleate desaturase (delta-12; OAory_01106610) were transcriptionally downregulated in the C6_t2 culture, which could be explained by feedback regulation of lipogenic enyzmes, as previously described [16]. Additionally, phosphomevalonate kinase (OAory_01041440), squalene monooxygenase (OAory_01020110), methylsterol monooxygenase (OAory_01043030 and OAory_01021800), and sterol 24-C-methyltransferase (OAory_01027770), which participated in the sterol biosynthesis pathway, were also transcriptionally downregulated at lipid-accumulating phase. In contrast, the expression of hydroxymethylglutaryl-CoA synthase 1 gene (*HMGCS1*; OAory_01041580) was upregulated in the C6_t2 culture. Functional analysis of the previous study exhibited the role of HMGCS in catalyzing the formation of hydroxymethylglutaryl-CoA from acety-CoA and acetoacetyl-CoA [52], which is a key link between primary metabolism and secondary metabolism, i.e., between the glycolysis pathway and the terpenoid backbone biosynthesis pathway. Thus, the upregulation in *HMGCS1* gene expression found in the glucose culture at lipid-accumulating phase was similar to the recent study highlighting that the respective enzyme was overexpressed to enhance the production of precursor substances from the mevalonate pathway [53]. Additionally, we found that the expression levels of several genes involved in amino acid metabolism decreased at the lipid-accumulating phase as compared to the C6_t1 culture, i.e., glutathione S-transferase (gst; OAory_01010430, OAory_01061170, and OAory_01076010), indoleamine 2,3-dioxygenase (ido; OAory_01102470), and 4-hydroxyphenylpyruvate dioxygenase (hpd; OAory_01064800), as shown in Table S4.

## 4. Discussion

A more systemic study in oleaginous fungi is required to better understand the carbon flux toward dynamic growth and lipid biosynthesis [12]. Mostly, biochemical events of lipid-accumulating processes in oleaginous filamentous fungi have been proposed in cultivations using glucose as a carbon source [17,32]. In this study, we explored how the fungal cells respond to xylose utilization toward lipid production through the comparative transcriptome and phenotypic analyses of *A. oryzae* BCC7051. The efficient growth ability of *A. oryzae* strain BCC7051 on xylose and glucose could be explained by transcriptional activation of unique sets of membrane transporters, hydrolytic enzymes, and other metabolic enzymes (Figure 2). The carbon uptake mechanism of the fungal cells was transcriptionally regulated in different manners depending on sugar type, seen through the upregulated DEGs in different groups of membrane transporters. The MFS transporter family and other ion transporters (aap, pot, ncs1) may play roles in xylose transport of *A. oryzae*. Conversely, another set of the membrane transporters, including glucose transporter (rco3) and ion transporters (cep, Catp, CaCA and Cacc), may cooperate in glucose uptake and transport of *A. oryzae*. It has been reported that glucose acts as a trigger for enhancing influx of calcium from the surrounding environment [54,55,56]. A high expression of the anion Cacc may be a responsive mechanism of *A. oryzae* cells, resulting in increased intracellular calcium concentration for glucose transport [57]. These results suggest that there were multiple transporters that played a role in carbon/nutrient uptake for cell growth and metabolism of *A. oryzae*. 

Similar to the previous reports of other filamentous fungi [58,59,60,61,62], xylanolytic enzymes of *A. oryzae* BCC7051 were transcripitonally upregulated using a xylose growth medium in contrast to the transcriptional response of the AmyR and amylolytic enzyme system in the glucose cultivation of *A. oryzae* BCC7051. The transcriptome results also reflect an interplay across several metabolic pathways, mostly in carbohydrate, amino acid, and lipid metabolisms (Figure 3) for leveraging cell growth and lipid production, which had different expression profiles not only between growth stages but also the sugars utilized. The overexpression of metabolic genes in the glycolysis found in the lipid-accumulating stage of the C5 culture under carbon-excess condition (Figure 4 and Table 1) indicated that the fungal cell had a robust metabolic control for balancing cofactors (NADPH or NADH) and ATP during xylose growth. The upregulated expression of ATP-citrate synthases in TCA cycle found in the C5 culture also points to a crucial link between energy and lipid metabolism through the metabolic flux from carbohydrates to fatty acid biosynthesis [63]. Additionally, the upregulation of the DEGs in the glycolysis pathway of the C5 culture might lead to an enhanced generation of pyruvate and acetyl-CoA, which are essential precursors for the TCA cycle and lipid biosynthesis, respectively, which coincides with the increase in biomass titer and total lipid content in a late logarithmic phase (Table 2). Moreover, its lipid productivity (Q_p_) was comparable to the C6 culture grown at the same stage (lipid-accumulating phase). It seems likely that the *A. oryzae* cells had reprogrammed its metabolic behavior for lipid production in the xylose cultivation in order to render oleaginous features.

Focusing on the metabolism of acetyl-CoA, it is a common precursor shared among the ergosterol and fatty acid biosynthetic pathways in mapping the transcript profiles of the C5 and C6 cultures of *A. oryzae* into its relevant metabolic networks (Figure 5). Upregulated DEGs in the sterol biosynthetic pathway are consistent with the high contents of TAG and SE of both C5 and C6 cultures grown at lipid-accumulating phase. With the existence of two HMGCS genes in the *A. oryzae* BCC7051 genome [3], it is noteworthy that HMGCS1 and HMGCS2 of *A. oryzae* BCC7051 played a role in functioning at the lipid-accumulating stage depending on sugar sources, in which HMGCS1 (OAory_01041580) expression was induced in the C6 culture, whereas the upregulated HMGCS2 (OAory_01061340) was found in the C5 culture. Therefore, these results provide compelling evidence that HMGSs were the key enzymes attributing in several metabolic processes, particularly in supplying the precursor for the mevalonate biosynthetic pathway in *A. oryzae* BCC705. The partition of acetyl-CoA molecule into the fatty acid biosynthetic pathway was also transcriptionally regulated. Concurring with the previous reports of the acetyl-CoA generation [12,64], the upregulated expression of a gene set, such as phosphoglycerate mutase, glutamate synthase, glutamate decarboxylase, and glutamate dehydrogenase (NADP^−^), might explain the fungal capability in increasing acetyl-CoA pool through amino acid metabolism for lipid storage during xylose cultivations. We also explored the involvement of amino acid metabolism in acetyl-CoA generation, which has been previously proposed by comparative genomic analysis of oleaginous yeasts and fungi [12]. The hydroxymethylglutaryl-CoA lyase (OAory_01034810) in the branched-chain amino acids (leucine) pathway in *A. oryzae* BCC7051 was transcriptionally controlled, which was significantly active in the xylose growth at lipid-acumulating phase. The relationship between the function of an alternative route for acetyl-CoA generation via amino acid metabolism and xylose utilization indicated that the hydroxymethylglutaryl-CoA lyase might be considered as key enzyme for oleagnicity of *A. oryzae*. In addition, other enzymes involved in amino acid metabolism, such as glutamate synthase (OAory_01081090), glutamate dehydrogenase (OAory_01030390), and aldehyde dehydrogenase (OAory_01017030), were transcriptionally upregulated, which might support the sufficient NADPH pool for fatty acid biosynthesis. Not only for providing energy and intermediate metabolites for celllular processes, the amino acid metabolism played a significant role in the lipid-accumulating process of *A. oryzae* when using xylose as a carbon source. 

This study suggested that the growth and metabolic behaviors of the oleaginous *A. oryzae* can be markedly impacted by accessible sugars in addition to other nutritional conditions (i.e., nitrogen sources, micronutrients, and C:N ratio). The oleaginicity features of *A. oryzae* on xylose growth was sustained through the sophisticated coordination of several metabolisms. In context to a circular economy, this meaningful information would guide to the development of microbial lipid production using cheap cellulosic sugar feedstocks from agroindustrial residues by adjusting carbon fluxes toward lipid biosynthesis through either genetic manipulation at key metabolic reactions or physiological control.

## 5. Conclusions

Taken together, *A. oryzae* BCC7051 had a robust metabolic control in lipid production of carbon sources (xylose and glucose) through at least transcriptional regulation. The plausible mechanisms of the oleaginous fungal cells in leveraging growth and lipid-accumulating capacity were discovered. Although the main metabolic pathways and biochemical regulation in lipid-accumulating process seems to be conserved among oleaginous strains, the discrimination in transcriptional control in lipid production of *A. oryzae* was sugar-dependent, particularly in amino acid metabolism, which played a role in maintaining oleaginous features and lipid homeostasis for normal growth on xylose in *A. oryzae*. This study provides remarkable insight in carbon utilization of *A. oryzae*, thereby contributing to lipogenesis in the filamentous fungi in relation to the growth performance that enables the exploitation of this fungal strain as a cell chassis for a cost-effective production of nutritionally important PUFAs and other oleochemicals using renewable carbon feedstocks through synthetic biology and bioprocessing approaches targeting fatty acid modification and relevant cellular metabolisms.

## Figures and Tables

**Table 1:** Kinetic parameters of cell growth and lipid production of the C5 and C6 cultures of *A. oryzae* BCC7051 grown at active growth (t1) and late logarithmic (t2) stages.

Parameters	C5 Cultures		C6 Cultures	
	C5_t1 ^a^	C5_t2 ^b^	C6_t1 ^c^	C6_t2 ^d^
DCW (g L^−1^)	7.07 ± 0.10 ^A^	11.66 ± 0.03 ^C^	7.48 ± 0.15 ^A^	10.85 ± 0.21 ^D^
Lipid content (% in DCW, *w*/*w*)	8.00 ± 2.55 ^A^	17.70 ± 0.42 ^C^	8.80 ± 0.28 ^A^	14.40 ± 0.00 ^D^
Residual sugar concentration (g L^−1^)	57.80 ± 0.00 ^A^	22.27 ± 0.08 ^C^	43.27 ± 0.12 ^B^	10.77 ± 0.00 ^D^
Q_X_ (g L^−1^ day^−1^)	4.36 ± 0.07 ^A^	2.78 ± 0.01 ^C^	4.63 ± 0.10 ^A^	3.44 ± 0.07 ^D^
Q_P_ (g L^−1^ day^−1^)	0.38 ± 0.13 ^A^	0.52 ± 0.01 ^C^	0.44 ± 0.02 ^A^	0.52 ± 0.01 ^C^
Q_S_ (mol L^−1^ day^−1^)	0.53 ± 0.00 ^A^	0.49 ± 0.00 ^C^	0.82 ± 0.00 ^B^	0.77 ± 0.00 ^D^
Y_X/S_ (g mol^−1^)	8.28 ± 0.13 ^A^	5.65 ± 0.02 ^C^	5.65 ± 0.12 ^B^	4.47 ± 0.00 ^D^
Y_P/S_ (g mol^−1^)	0.72 ± 0.04 ^A^	1.05 ± 0.03 ^C^	0.53 ± 0.03 ^B^	0.68 ± 0.01 ^D^

All data are represented as the mean of values with standard deviation (SD), which were derived from independently triplicate experiments. Values marked with different superscript letters A,B in the same row of the C5 and C6 cultures at the active growth stage (C5_t1 vs. C6_t1) are significantly different (*p* < 0.05). Values marked with different superscript letters C,D in the same row of the C5 and C6 cultures at late logarithmic stage (C5_t2 vs. C6_t2) are significantly different (*p* < 0.05). Superscript letters a,b indicate the values calculated from the C5 cultures cultivated for 0–36 h and 0–96 h, respectively. Superscript letters c,d indicate the values calculated from the C6 cultures cultivated for 0–36 h and 0–72 h, respectively.

**Table 2:** Proportional composition of lipid classes of the C5 and C6 cultures of *A. oryzae* BCC7051 grown at active growth (t1) and late logarithmic (t2) stages.

Proportion in Total Lipid (% *w*/*w*)	C5 Cultures		C6 Cultures	
	C5_t1 ^a^	C5_t2 ^b^	C6_t1 ^c^	C6_t2 ^d^
SE	12.0 ± 1.1 ^A^	13.5 ± 1.7 ^C^	11.6 ± 0.6 ^A^	9.2 ± 1.6 ^D^
TAG	30.7 ± 0.2 ^A^	34.9 ± 1.7 ^C^	30.9 ± 2.6 ^A^	34.7 ± 4.0 ^C^
FFA	28.2 ± 0.7 ^A^	23.1 ± 2.6 ^C^	31.3 ± 1.7 ^B^	28.9 ± 1.9 ^D^
PL	5.3 ± 0.2 ^A^	5.6 ± 0.1 ^C^	5.1 ± 0.4 ^A^	5.6 ± 0.3 ^C^
Others	23.8 ± 0.8 ^A^	22.9 ± 2.6 ^C^	21.1 ± 1.9 ^A^	21.6 ± 4.1 ^C^

All data are represented as the mean of values with standard deviation (SD), which were derived from independently triplicate experiments. ^A,B^ Values marked with different superscript letters in the same row of active growth cultures using different sugars (C5_t1 vs. C6_t1) are significantly different (*p* < 0.05). ^C,D^ Values marked with different superscript letters in the same row of late logarithmic cultures using different sugars (C5_t2 vs. C6_t2) are significantly different (*p* < 0.05). Superscript letters a,b indicate mean values derived from the C5 cultures grown for 36 and 96 h, respectively. Superscript letters c,d indicate mean values derived from the C6 cultures grown for 36 and 72 h, respectively.

**Table 3:** Fatty acid composition in total lipid of the C5 and C6 cultures of *A. oryzae* BCC7051 grown at active growth (t1) and late logarithmic (t2) stages.

Proportion in Total Fatty Acid (% *w*/*w*)	C5 Cultures		C6 Cultures	
	C5_t1 ^a^	C5_t2 ^b^	C6_t1 ^c^	C6_t2 ^d^
C14:0	0.0 ± 0.0 ^A^	0.2 ± 0.0 ^C^	0.0 ± 0.0 ^A^	0.0 ± 0.0 ^C^
C16:0	15.9 ± 0.6 ^A^	15.9 ± 0.1 ^C^	13.4 ± 0.0 ^B^	14.1 ± 0.0 ^D^
C16:1Δ^9^	0.9 ± 0.0 ^A^	0.4 ± 0.0 ^C^	0.0 ± 0.0 ^B^	0.0 ± 0.0 ^C^
C18:0	13.3 ± 0.1 ^A^	18.7 ± 0.1 ^C^	11.6 ± 0.2 ^B^	16.1 ± 0.4 ^D^
C18:1Δ^9^	28.9 ± 0.2 ^A^	33.5 ± 0.2 ^C^	25.9 ± 0.1 ^B^	29.9 ± 0.4 ^D^
C18:2Δ^9,12^	40.1 ± 0.3 ^A^	29.8 ± 0.0 ^C^	48.5 ± 0.3 ^B^	39.0 ± 0.7 ^D^
C18:3Δ^9,12,15^	0.0 ± 0.0 ^A^	0.0 ± 0.0 ^C^	0.0 ± 0.0 ^A^	0.0 ± 0.0 ^C^
C20:0	0.9 ± 0.0 ^A^	1.5 ± 0.0 ^C^	0.6 ± 0.0 ^A^	0.9 ± 0.2 ^D^

All data are represented as the mean of values with standard deviation (SD), which were derived from independently triplicate experiments. ^A,B^ Values marked with different superscript letters in the same row of active growth cultures using different sugars (C5_t1 vs. C6_t1) are significantly different (*p* < 0.05). ^C,D^ Values marked with different superscript letters in the same row of late logarithmic cultures using different sugars (C5_t2 vs. C6_t2) are significantly different (*p* < 0.05). Superscript letters a,b indicate mean values derived from the C5 cultures grown for 36 and 96 h, respectively. Superscript letters c,d indicate mean values derived from the C6 cultures grown for 36 and 72 h, respectively.

## Data Availability

Raw read data has been submitted to NCBI Sequence Read Archive (SRR14929132–SRR14929135).

## Supplementary Materials

The following are available online at https://www.mdpi.com/article/10.3390/biology10090885/s1, Figure S1: Growth profiles of *A. oryzae* cultures grown in SM media containing 80 g of xylose (A) and glucose (B) at 30 °C with agitation rate of 500 rpm, air flow rate of 1.0 vvm, and pH at 4.5, Figure S2: The number of gene expression levels with FPKM > 1 of the *A. oryzae* cultures grown at different conditions. C5_t1 and C5_t2 indicate the 36 h and 96 h cultures of *A. oryzae* using xylose as a sole carbon source, respectively. C6_t1 and C6_t2 indicate the glucose cultures of *A. oryzae* grown for 36 and 72 h, respectively, Table S1: Transcriptome datasets of *A. oryzae* cultures grown on xylose and glucose at different growth stages. The C5_t1 and C5_t2 samples indicate the xylose cultures grown for 36 and 96 h, respectively. The C6_t1 and C6_t2 samples represent the glucose cultures grown for 36 and 72 h, respectively, Table S2: Lists of consensus up- and downregulated genes in *A. oryzae* BCC7051 cultures using xylose and glucose, respectively, Table S3: Gene expression levels of hydroxymethylglutaryl-coenzyme A reductases (*hmg*) of the *A. oryzae* cultures using xylose (C5_t1 and C5_t2) and glucose (C6_t1 and C6_t2), Table S4: Expression level of genes involved in the carbohydrate, lipid, and amino acid metabolic pathways of *A. oryzae* BCC7051.
