# Supplementary material for: Metabolic Regulation of Sugar Assimilation for Lipid Production in Aspergillus oryzae BCC7051 through Comparative Transcriptome Perspective
Source: Biology (Basel). 2021 Sep 8;10(9):885. doi: 10.3390/biology10090885 (PMC8467706; doi:10.3390/biology10090885)
Supplement: Supplementary file 1 [file biology-10-00885-s001.zip › biology-1318615-supplementary.pdf]

biology

MDPI

Article

Metabolic Regulation of Sugar Assimilation for Lipid Production in *Aspergillus oryzae* BCC7051 through Comparative Transcriptome Perspective

Tayvich Vorapreeda [1], Bhimabol Khongto [2], Chinae Thammarongtham [1], Tanawut Srisuk [3] and Kobkul Laoteng [2,*]

[1] Biochemical Engineering and Systems Biology Research Group, National Center for Genetic Engineering and Biotechnology (BIOTEC), National Science and Technology Development Agency (NSTDA), at King Mongkut's University of Technology Thonburi, Bangkok 10150, Thailand; tayvich.vor@biotec.or.th (T.V.); chinae@biotec.or.th (C.T.)
[2] Functional Ingredients and Food Innovation Research Group, National Center for Genetic Engineering and Biotechnology (BIOTEC), National Science and Technology Development Agency (NSTDA), Thailand Science Park, Pathum Thani 12120, Thailand; bhimabol.kho@biotec.or.th (B.K.)
[3] Pilot Plant Development and Training Institute, King Mongkut's University of Technology Thonburi, Bangkok 10150, Thailand; tanawut.sri@mail.kmutt.ac.th (T.S.)
* E-mail address: kobkul@biotec.or.th

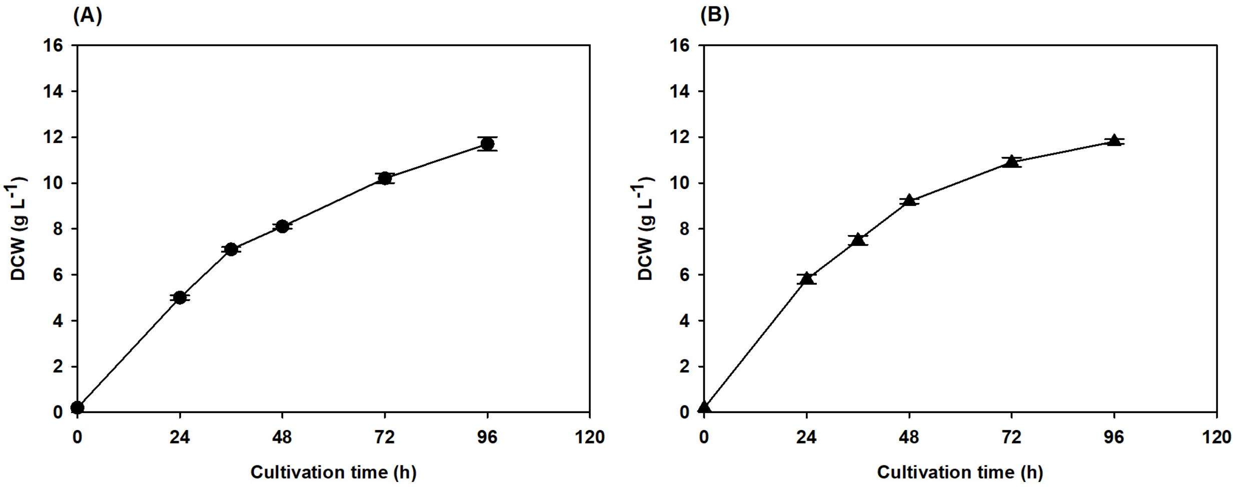

Figure S1: Growth profiles of *A. oryzae* cultures grown in SM media containing 80 g of xylose (A) and glucose (B) at 30°C with agitation rate of 500 rpm, air flow rate of 1.0 vvm and pH at 4.5.

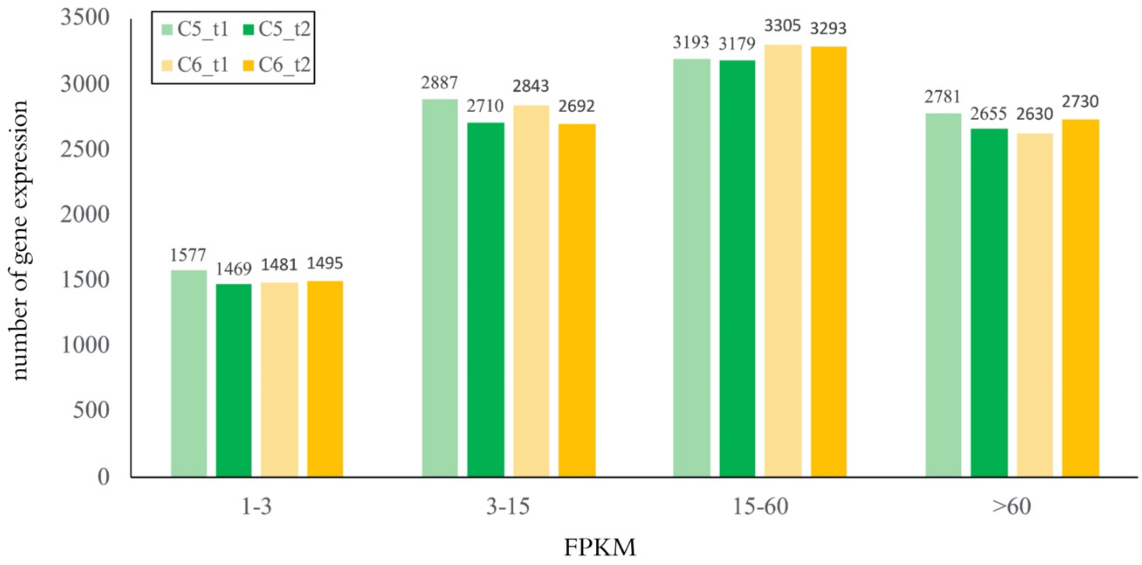

Figure S2: The number of gene expression levels with FPKM >1 of the *A. oryzae* cultures grown at different conditions. C5_t1 and C5_t2 indicate the 36-h and 96-h cultures of *A. oryzae* using xylose as a sole carbon source, respectively. C6_t1 and C6_t2 indicate the glucose cultures of *A. oryzae* grown for 36 and 72 h, respectively.

Table S1: Transcriptome datasets of *A. oryzae* cultures grown on xylose and glucose at different growth stages. The C5_t1 and C5_t2 samples indicate the xylose cultures grown for 36 and 96 h, respectively. The C6_t1 and C6_t2 samples represent the glucose cultures grown for 36 and 72 h, respectively.

Sample	No. of raw reads (bp)	No. of high quality reads (bp)	Total mapped reads (bp)	Q30 (%)	GC content (%)
C5_t1	43,183,728	38,021,532	33,857,973	92.57	49.78
C5_t2	42,786,228	37,989,668	33,890,233	92.39	51.47
C6_t1	40,052,840	36,972,478	33,073,489	93.55	50.85
C6_t2	50,530,010	47,067,126	42,539,108	92.66	51.52

Table S2: Lists of consensus up- and downregulated genes in *A. oryzae* BCC7051 cultures using xylose and glucose, respectively.

				FPKM				
	**Gen_ID**	**Locus_tag**	**Gene_descrption**	**C5_t1**	**C5_t2**	**C6_t1**	**C6_t2**	**symbol**
Consensus up-regulated genes in *A. oryzae* BCC7051 cultured with xylose	Novel00433	--	hypothetical protein	162.969	391.922	17.271	14.644	
	gene10186	OAory_01109210	alpha-xylosidase	127.599	168.003	4.791	2.990	xyl1
	gene10228	OAory_01109630	flavin-nucleotide-binding protein; K07005	137.736	103.847	54.631	52.310	
	gene10625	OAory_01113600	short chain oxidoreductase/dehydrogenase	112.615	63.928	9.014	18.170	
	gene11451	OAory_01072900	hypothetical protein	141.838	174.135	30.232	27.715	
	gene136	OAory_01024140	O-methyltransferase	26.360	65.032	4.216	5.011	
	gene137	OAory_01024150	MFS superfamily	41.397	89.949	5.343	4.557	mfs-x4
	gene1720	OAory_01001840	aldo/keto reductase	343.552	194.698	63.804	90.925	
	gene2013	OAory_01004770	L-arabitol dehydrogenase; K00008 L-iditol 2-dehydrogenase [EC:1.1.1.14]	157.144	99.577	26.875	34.672	sorD
	gene202	OAory_01024800	FAD dependent oxidoreductase superfamily	95.063	122.311	33.801	43.499	
	gene2086	OAory_01005500	exo-1,4-beta-xylosidase xlnD; K15920 beta-D-xylosidase 4 [EC:3.2.1.37]	89.503	34.993	4.857	5.995	xylA
	gene2281	OAory_01007450	alpha-D-xyloside xylohydrolase [EC:3.2.1.177]	28.228	20.772	0.336	0.185	xyl2
	gene2337	OAory_01008010	glycosyl hydrolase family 43; α-L-arabinofuranosidases	246.756	98.819	9.009	6.737	
	gene2461	OAory_01009250	hypothetical protein	43.676	54.048	2.681	5.512	
	gene2770	OAory_01012340	hypothetical protein	3530.213	5523.282	1549.735	382.012	
	gene3452	OAory_01077770	hypothetical protein	30.243	45.427	5.604	5.574	
	gene4277	OAory_01086020	hypothetical protein	550.546	287.917	154.967	139.465	
	gene4287	OAory_01086120	aldo-keto reductase yakc [NADP+]; oxidoreductase	464.853	472.630	92.926	98.684	
	gene4414	OAory_01053690	integral membrane protein TmpA	109.684	70.646	34.693	26.464	
	gene4551	OAory_01055060	endoglucanase A; K18576 xyloglucan-specific endo-beta-1,4-glucanase [EC:3.2.1.151]	14.840	21.690	0.106	0.078	xeg
	gene4971	OAory_01059260	NAD(P)H-dependent D-xylose reductase xyl1; K17743 D-xylose reductase [EC:1.1.1.307]	2682.455	2308.642	23.049	25.897	xr1
	gene5035	OAory_01059900	MFS sugar transporter	205.212	119.827	5.745	6.068	mfs-x1
	gene5036	OAory_01059910	alcohol dehydrogenase	96.441	36.195	7.795	8.938	
	gene5342	OAory_01062970	hypothetical protein	78.629	147.097	2.075	2.286	
	gene5343	OAory_01062980	flavo protein	96.825	79.687	3.868	3.653	
	gene5402	OAory_01063570	Copper amine oxidase domain-containing protein	56.533	64.712	18.196	24.515	
	gene5472	OAory_01064270	nucleoside transporter	71.042	182.008	31.756	29.382	ncs1
	gene5723	OAory_01096310	hypothetical protein	878.832	860.281	229.197	297.293	
	gene5730	OAory_01096380	hypothetical protein	63.210	28.440	4.009	3.303	
	gene5736	OAory_01096440	D-xylulose reductase A; K05351 D-xylulose reductase [EC:1.1.1.9]	1148.227	1663.494	12.874	21.005	xdh1
	gene5741	OAory_01096490	extracellular invertase; Glycosyl hydrolase family	23.482	116.583	6.436	3.293	
	gene5774	OAory_01096820	D-xylulose kinase A; K00854 xylulokinase [EC:2.7.1.17]	184.513	348.891	10.759	17.285	xylB
	gene6257*	OAory_01101650	aldose 1-epimerase; K01785 aldose 1-epimerase [EC:5.1.3.3]	200.568	339.276	35.337	24.670	galM
	gene6299	OAory_01102070	D-xylulose reductase A; K05351 D-xylulose reductase [EC:1.1.1.9]	867.282	946.985	88.237	103.257	xdh2
	gene6469	OAory_01103770	alpha-xylosidase	33.823	34.358	4.263	3.743	xyl3
	gene6809	OAory_01107170	MFS multidrug transporter	128.180	118.230	8.539	5.987	mfs-x2
	gene6876	OAory_01107840	extracellular proline-serine rich protein	46.416	42.314	15.944	5.295	
	gene6877	OAory_01107850	MFS monosaccharide transporter	256.286	62.484	11.106	12.950	mfs-x3
	gene6890	OAory_01107980	short-chain oxidoreductase	85.533	62.632	5.734	8.983	
	gene6920	OAory_01108280	Non-heme dioxygenase N-terminal domain	140.402	143.811	3.544	60.139	
	gene6921	OAory_01108290	oligopeptide transporter; POT family	47.832	148.280	4.786	31.866	pot
	gene6988	OAory_01038710	amino acid permease	346.837	316.439	66.903	155.966	aap
	gene7298	OAory_01041810	oxidoreductase	33.449	326.090	3.653	3.238	
	gene8036	OAory_01088530	cipC protein	357.575	727.547	132.720	161.321	
	gene8096	OAory_01089130	short-chain dehydrogenase/reductase family protein	72.165	53.446	4.672	5.115	
	gene8969	OAory_01017000	hypothetical protein	55.417	30.964	4.439	2.690	
	gene9009	OAory_01017400	AO090701000360; 1-aminocyclopropane-1-carboxylate deaminase; K01505 1-aminocyclopropane-1-carboxylate deaminase [EC:3.5.99.7]	677.811	591.877	81.470	94.458	accD
	gene9382	OAory_01021130	AO090701000824; oxidoreductase	205.093	128.320	74.164	55.878	
	gene9430	OAory_01021610	AO090701000885; alpha-L-arabinofuranosidase axhA	33.189	18.250	0.837	0.553	axhA
	gene9938	OAory_01071200	AO090120000106; cytochrome P450 monooxygenase	45.788	42.705	15.860	16.406	

Consensus down-regulated genes in *A. oryzae* BCC7051 cultured with glucose	Novel00241	--	transposase	141.346	27.548	357.233	431.208	
	Novel00354	--	transposase	24.598	4.523	45.761	45.829	
	Novel00356	--	--	15.221	7.214	44.375	23.444	
	Novel00938	--	ABC multidrug transporter	33.553	27.084	98.819	114.281	abc1
	gene10068	OAory_01072500	calcium/proton antiporter, CaCA family	27.820	51.107	61.866	155.449	CaCA
	gene10069	OAory_01072510	plasma membrane calcium-transporting ATPase	15.351	25.178	37.011	61.930	Catp
	gene10670	OAory_01114050	deoxyribose-phosphate aldolase; K01619 deoxyribose-phosphate aldolase [EC:4.1.2.4]	1.334	0.492	46.955	20.907	deoC
	gene10837	OAory_01049080	malate synthase, glyoxysomal; K01638 malate synthase [EC:2.3.3.9]	99.809	55.403	340.298	183.408	aceB
	gene11212	OAory_01052830	hypothetical protein	237.614	279.957	1953.346	1005.375	
	gene1359	OAory_01036370	C2H2 finger domain protein FlbC	48.885	55.167	103.961	125.430	
	gene1420	OAory_01036980	flavonoid 3-hydroxylase; cytochrome P450	12.854	5.757	34.563	31.174	
	gene1435	OAory_01037130	endo mannanase, GH76 family	35.784	38.848	201.032	208.977	
	gene1436	OAory_01037140	hypothetical protein	2.627	2.856	27.025	40.008	
	gene1930	OAory_01003940	hypothetical protein	13.947	6.280	79.250	70.092	
	gene2307	OAory_01007710	HAD-like domain-containing protein	251.296	161.099	527.776	474.049	
	gene2684	OAory_01011480	hypothetical protein	120.810	71.608	259.337	533.488	
	gene2850	OAory_01013140	oxalate decarboxylase	19.062	11.247	63.731	70.829	
	gene2852	OAory_01013160	sugar transporter / glucose transporter rco-3	109.780	34.358	464.548	306.572	rco3
	gene295	OAory_01025730	AMP dependent CoA ligase	9.807	1.349	41.979	21.200	
	gene2997	OAory_01073220	beta-lactamase family protein	48.372	0.658	214.936	96.841	lactB
	gene2998	OAory_01073230	hypothetical protein	90.547	0.927	361.244	194.271	
	gene3024	OAory_01073490	Cytochrome P450	3.598	3.086	48.921	138.950	
	gene3092	OAory_01074170	C6 transcription factor	0.674	3.119	6.866	492.457	C6-tf
	gene3111	OAory_01074360	hypothetical protein	0.331	1.757	7.685	265.352	
	gene371	OAory_01026490	hypothetical protein	1176.713	638.744	4072.493	3011.633	
	gene4043	OAory_01083680	peroxidase	58.468	30.510	121.205	129.440	
	gene4093	OAory_01084180	cation efflux protein	66.138	26.873	159.467	110.359	cep
	gene4686	OAory_01056410	alpha-amylase A type-1/2	4.135	2.319	57.850	59.343	amy1
	gene4687	OAory_01056420	alpha-glucosidase; K01187 alpha-glucosidase [EC:3.2.1.20]	9.479	10.741	39.049	43.417	malZ
	gene4688	OAory_01056430	amylase cluster transcriptional regulator AmyR	12.513	11.287	29.569	37.050	amyR
	gene4784	OAory_01057390	fructose-1,6-bisphosphatase; K03841 fructose-1,6-bisphosphatase I [EC:3.1.3.11]	55.945	42.345	240.195	103.333	fbp
	gene4825	OAory_01057800	oryzin; K18549 oryzin [EC:3.4.21.63]	13.189	23.600	50.683	96.509	
	gene5487	OAory_01064420	hydrolase	51.080	311.952	132.066	1893.196	
	gene5490	OAory_01064450	HSCARG dehydrogenase	166.911	737.622	330.597	3169.289	
	gene5546	OAory_01065010	phosphoenolpyruvate carboxykinase [ATP]; K01610 phosphoenolpyruvate carboxykinase (ATP) [EC:4.1.1.49]	34.108	43.906	159.130	99.380	pckA
	gene5808	OAory_01097160	alpha-amylase A type-1/2; K01176 alpha-amylase [EC:3.2.1.1]	2.271	0.618	52.016	59.707	amy2
	gene5877	OAory_01097850	hypothetical protein	18318.679	10422.264	34310.598	31442.565	
	gene66	OAory_01023440	NRPS-like enzyme	0.965	4.978	6.542	14.107	
	gene7163	OAory_01040460	polyamine oxidase; K13366 polyamine oxidase [EC:1.5.3.14 1.5.3.16 1.5.3.-]	41.213	48.534	81.168	135.635	pao
	gene7491	OAory_01043740	glucoamylase; K01178 glucoamylase [EC:3.2.1.3]	8.581	6.186	72.631	47.686	sga
	gene7588	OAory_01044710	GMC oxidoreductase	20.477	6.542	43.666	26.938	gmc
	gene7751	OAory_01046340	hypothetical protein	14.148	9.653	39.902	44.321	
	gene7752	OAory_01046350	hypothetical protein	44.204	15.621	184.808	165.783	
	gene7753	OAory_01046360	hypothetical protein	25.857	17.411	78.473	96.457	
	gene7813	OAory_01046960	hypothetical protein	153.721	206.150	382.833	533.814	
	gene7871	OAory_01047540	hypothetical protein	177.752	10.466	476.757	312.700	
	gene7872	OAory_01047550	AO090103000450; 2OG-Fe(II) oxygenase family oxidoreductase	354.522	9.936	670.955	541.550	
	gene8044	OAory_01088610	hypothetical protein	65.232	64.522	229.132	424.458	
	gene8061	OAory_01088780	calcium-activated chloride channel-domain-containing protein	4.906	12.499	16.378	30.950	Cacc
	gene8305	OAory_01091220	hypothetical protein	6.479	6.722	37.094	140.556	
	gene8314	OAory_01091310	fructosyl amine:oxygen oxidoreductase	29.770	23.053	64.176	121.818	fao
	gene8515	OAory_01093320	hypothetical protein	238.511	143.297	918.021	479.119	
	gene9057	OAory_01017880	cytochrome P450	87.297	53.982	171.343	266.135	
	gene9161	OAory_01018920	hypothetical protein	615.858	153.418	1533.226	519.192	
	gene9225	OAory_01019560	protein alcS	12.392	0.537	57.664	28.815	
	gene9244	OAory_01019750	hypothetical protein	34.929	29.179	72.851	68.408	
	gene9245	OAory_01019760	hmg-CoA reductase	16.998	9.714	60.518	81.696	hmgR1
	gene9677	OAory_01068590	hypothetical protein	106.468	114.838	403.174	246.019	
	gene9767	OAory_01069490	C6 zinc finger domain protein	60.436	55.154	111.794	116.909	

Table S3: Gene expression level of hydroxymethylglutaryl-coenzyme A reductases (*hmgR*) of *A. oryzae* cultures using xylose (C5_t1 and C5_t2) and glucose (C6_t1 and C6_t2).

		FPKM			
**Locus_tag**	**Symbol**	**C5_t1**	**C5_t2**	**C6_t1**	**C6_t2**
OAory_01019760	*hmgR1*	17.00	9.71	60.52	81.70
OAory_01046250	*hmgR2*	0.00	0.14	0.00	0.16
OAory_01111690	*hmgR3*	3.72	6.16	3.29	3.46

Table S4: Expression level of genes involved in the carbohydrate, lipid and amino acid metabolic pathways of *A. oryzae* BCC7051.

(a) glycolysis pahway

Locus_tag	Gene_ID	Description	EC	C5_t1_fpkm	C5_t2_fpkm	C6_t1_fpkm	C6_t2_fpkm	C5_t1(log2fpkm+1)	C5_t2(log2fpkm+1)	C6_t1(log2fpkm+1)	C6_t2(log2fpkm+1)	FC_C5	FC_C6
OAory_01107650	gene6857	phosphoglucomutase	5.4.2.2	49.67	37.86	35.86	36.14	5.66	5.28	5.20	5.21	0.76	1.01
OAory_01060200	gene5065	phosphoglucomutase	5.4.2.2	341.71	370.26	287.94	338.27	8.42	8.54	8.17	8.41	1.08	1.17
OAory_01092830	gene8466	glucose 6-phosphate isomerase	5.3.1.9	238.62	434.49	204.97	212.92	7.90	8.77	7.69	7.74	1.82	1.04
OAory_01013110	gene2847	hexokinase	2.7.1.1	10.25	12.56	18.08	20.95	3.49	3.76	4.25	4.46	1.22	1.16
OAory_01079580	gene3633	aldose 1-epimerase	5.1.3.3	17.01	10.40	15.14	11.96	4.17	3.51	4.01	3.70	0.61	0.79
OAory_01101650	gene6257	aldose 1-epimerase	5.1.3.3	200.57	339.28	35.34	24.67	7.66	8.41	5.18	4.68	1.69	0.70
OAory_01057390	gene4784	fructose-1,6-bisphosphatase	3.1.3.11	55.94	42.34	240.19	103.33	5.83	5.44	7.91	6.71	0.76	0.43
OAory_01041240	gene7241	6-phosphofructokinase	2.7.1.11	118.16	414.19	132.43	48.08	6.90	8.70	7.06	5.62	3.51	0.36
OAory_01060380	gene5083	fructose-bisphosphate aldolase	4.1.2.13	928.63	1523.41	1945.16	1116.74	9.86	10.57	10.93	10.13	1.64	0.57
OAory_01041800	gene7297	fructose-bisphosphate aldolase	4.1.2.13	12.78	134.14	3.45	3.03	3.78	7.08	2.15	2.01	10.49	0.88
OAory_01033650	gene1087	triosephosphate isomerase	5.3.1.1	420.37	325.62	334.11	239.72	8.72	8.35	8.39	7.91	0.77	0.72
OAory_01068080	gene9626	triosephosphate isomerase	5.3.1.1	465.42	816.93	383.23	479.80	8.87	9.68	8.59	8.91	1.76	1.25
OAory_01055530	gene4598	glyceraldehyde 3-phosphate dehydrogenase	1.2.1.12	1584.89	6039.78	3311.43	2813.27	10.63	12.56	11.69	11.46	3.81	0.85
OAory_01090800	gene8263	glyceraldehyde 3-phosphate dehydrogenase	1.2.1.12	13425.72	13385.92	10179.58	13550.39	13.71	13.71	13.31	13.73	1.00	1.33
OAory_01104040	gene6496	phosphoglycerate kinase	2.7.2.3	535.54	1822.49	973.46	675.32	9.07	10.83	9.93	9.40	2.75	0.69
OAory_01003010	gene1837	phosphoglycerate mutase	5.4.2.12	242.22	666.38	360.84	294.30	7.93	9.38	8.50	8.21	2.20	0.82
OAory_01070940	gene9912	phosphoglycerate mutase	5.4.2.12	233.91	515.70	330.37	180.14	7.88	9.01	8.37	7.50	2.20	0.55
OAory_01065890	gene5634	enolase	4.2.1.11	571.41	1134.59	1171.39	940.13	9.16	10.15	10.20	9.88	1.99	0.80
OAory_01000800	gene1616	pyruvate kinase	2.7.1.40	276.98	597.77	377.99	476.54	8.12	9.23	8.57	8.90	2.16	1.26
OAory_01065010	gene5546	phosphoenolpyruvate carboxykinase (ATP)	4.1.1.49	34.11	43.91	159.13	99.38	5.13	5.49	7.32	6.65	1.29	0.62
OAory_01064060	gene5451	pyruvate dehydrogenase E1 component alpha subu	1.2.4.1	11.02	40.49	64.65	16.26	3.59	5.37	6.04	4.11	3.68	0.25
OAory_01060940	gene5139	pyruvate decarboxylase	4.1.1.1	422.38	5749.33	1517.39	411.75	8.73	12.49	10.57	8.69	13.61	0.27
OAory_01072460	gene10064	pyruvate decarboxylase	4.1.1.1	60.88	44.26	37.79	56.24	5.95	5.50	5.28	5.84	0.73	1.49
OAory_01091400	gene8233	dihydrolipoamide dehydrogenase	1.8.1.4	181.40	252.82	180.47	185.14	7.51	7.99	7.50	7.54	1.39	1.03
OAory_01010240	gene2560	pyruvate dehydrogenase E2 component	2.3.1.12	214.55	258.54	210.09	200.25	7.75	8.02	7.72	7.65	1.21	0.95
OAory_01057220	gene4767	acetyl-CoA synthetase	6.2.1.1	289.40	166.29	437.92	368.52	8.18	7.39	8.78	8.53	0.57	0.84
OAory_01017030	gene8972	aldehyde dehydrogenase (NAD+)	1.2.1.3	82.49	376.06	97.07	73.08	6.38	8.56	6.62	6.21	4.56	0.75
OAory_01026520	gene374	aldehyde dehydrogenase (NAD+)	1.2.1.3	426.53	123.20	549.10	749.56	8.74	6.96	9.10	9.55	0.29	1.37
OAory_01048490	gene10778	alcohol dehydrogenase, propanol-preferring	1.1.1.1	385.08	3173.09	1269.67	437.76	8.59	11.63	10.31	8.78	8.24	0.34
OAory_01112540	gene10519	alcohol dehydrogenase, propanol-preferring	1.1.1.1	114.99	12.42	113.33	66.67	6.86	3.75	6.84	6.08	0.11	0.59
OAory_01012690	gene2805	alcohol dehydrogenase, propanol-preferring	1.1.1.1	8.09	8.92	18.15	13.99	3.18	3.31	4.26	3.91	1.10	0.77

(b) TCA cycle

Locus_tag	Gene_ID	Description	EC	C5_t1_fpkm	C5_t2_fpkm	C6_t1_fpkm	C6_t2_fpkm	C5_t1(log2fpkm+1)	C5_t2(log2fpkm+1)	C6_t1(log2fpkm+1)	C6_t2(log2fpkm+1)	FC_C5	FC_C6
OAory_01065010	gene5546	phosphoenolpyruvate carboxykinase (ATP)	4.1.1.49	34.11	43.91	159.13	99.38	5.13	5.49	7.32	6.65	1.29	0.62
OAory_01010240	gene2560	pyruvate dehydrogenase E2 component	2.3.1.12	214.55	258.54	210.09	200.25	7.75	8.02	7.72	7.65	1.21	0.95
OAory_01064060	gene5451	pyruvate dehydrogenase E1 component alpha subunit	1.2.4.1	11.02	40.49	64.65	16.26	3.59	5.37	6.04	4.11	3.68	0.25
OAory_01091400	gene8233	dihydrolipoamide dehydrogenase	1.8.1.4	35.51	26.82	24.10	29.20	5.19	4.80	4.65	4.92	0.76	1.21
OAory_01029250	gene647	pyruvate carboxylase	6.4.1.1	148.29	123.75	151.48	189.33	7.22	6.96	7.25	7.57	0.83	1.25
OAory_01010220	gene2558	malate dehydrogenase	1.1.1.37	340.02	425.90	348.50	372.71	8.41	8.74	8.45	8.55	1.25	1.07
OAory_01014400	gene8709	malate dehydrogenase	1.1.1.37	919.97	1050.29	1295.84	1316.79	9.85	10.04	10.34	10.36	1.14	1.02
OAory_01071020	gene9920	fumarate hydratase	4.2.1.2	87.14	123.88	101.63	98.20	6.46	6.96	6.68	6.63	1.42	0.97
OAory_01041740	gene7291	succinate dehydrogenase	1.3.5.1	371.64	380.44	446.36	341.51	8.54	8.58	8.81	8.42	1.02	0.77
OAory_01022110	gene11386	succinyl-CoA synthetase	6.2.1.4/6.2.1.5	383.14	311.16	336.97	393.65	8.59	8.29	8.40	8.62	0.81	1.17
OAory_01101940	gene6286	2-oxoglutarate dehydrogenase E2 component	2.3.1.61	263.62	304.97	216.16	305.22	8.05	8.26	7.76	8.26	1.16	1.41
OAory_01004070	gene1943	2-oxoglutarate dehydrogenase, E2 subunit, dihydrolipoa	2.3.1.61	2.99	3.61	3.06	2.92	2.00	2.20	2.02	1.97	1.21	0.96
OAory_01057660	gene4811	2-oxoglutarate dehydrogenase, E1 subunit	1.2.4.2	66.76	87.11	80.98	79.08	6.08	6.46	6.36	6.32	1.30	0.98
OAory_01066230	gene5668	isocitrate dehydrogenase, NAD-dependent	1.1.1.41	376.35	526.10	424.98	400.69	8.56	9.04	8.73	8.65	1.40	0.94
OAory_01002200	gene1756	isocitrate dehydrogenase, NADP-dependent	1.1.1.42	117.64	107.32	142.67	127.70	6.89	6.76	7.17	7.01	1.41	0.90
OAory_01063070	gene5352	aconitate hydratase	4.2.1.3	195.08	275.95	378.40	330.13	7.62	8.11	8.57	8.37	0.06	0.87
OAory_01039610	gene7078	citrate synthase	2.3.3.1	1.88	0.12	0.12	1.22	1.53	0.16	0.17	1.15	0.06	9.91
OAory_01024350	gene157	ATP-citrate synthase subunit 2	2.3.3.8	374.93	829.81	384.90	274.26	8.55	9.70	8.59	8.10	2.21	0.71
OAory_01024360	gene158	ATP-citrate synthase subunit 1	2.3.3.8	234.45	677.44	271.91	196.62	7.88	9.41	8.09	7.63	2.89	0.72

c) pentose phosphate pathway

Locus_tag	Gene_ID	Description	EC	C5_t1_fpkm	C5_t2_fpkm	C6_t1_fpkm	C6_t2_fpkm	C5_t1(log2fpkm+1)	C5_t2(log2fpkm+1)	C6_t1(log2fpkm+1)	C6_t2(log2fpkm+1)	FC_C5	FC_C6
OAory_01092830	gene8466	glucose 6-phosphate isomerase	5.3.1.9	238.62	434.49	204.97	212.92	7.90	8.77	7.69	7.74	1.82	1.04
OAory_01057390	gene4784	fructose-1,6-bisphosphatase	3.1.3.11	55.94	42.34	240.19	103.33	5.83	5.44	7.91	6.71	0.76	0.43
OAory_01013110	gene2847	hexokinase	2.7.1.1	10.25	12.56	18.08	20.95	3.49	3.76	4.25	4.46	1.22	1.16
OAory_01060380	gene5083	fructose-bisphosphate aldolase	4.1.2.13	928.63	1523.41	1945.16	1116.74	9.86	10.57	10.93	10.13	1.64	0.57
OAory_01041800	gene7297	fructose-bisphosphate aldolase	4.1.2.13	12.78	134.14	3.45	3.03	3.78	7.08	2.15	2.01	10.49	0.88
OAory_01002030	gene1739	glucose-6-phosphate 1-dehydrogenase	1.1.1.49	428.56	555.97	366.13	408.91	8.75	9.12	8.52	8.68	1.30	1.12
OAory_01007270	gene2263	6-phosphogluconolactonase	3.1.1.31	121.92	153.26	123.69	131.87	6.94	7.27	6.96	7.05	1.26	1.07
OAory_01065380	gene5583	6-phosphogluconate dehydrogenase	1.1.1.44	411.92	529.12	387.40	438.76	8.69	9.05	8.60	8.78	1.28	1.13
OAory_01110120	gene10277	6-phosphogluconate dehydrogenase	1.1.1.44	0.06	2.29	4.68	0.98	0.08	1.72	2.51	0.98	40.71	0.21
OAory_01075460	gene3221	ribulose-phosphate 3-epimerase	5.1.3.1	132.01	119.07	101.17	106.01	7.06	6.91	6.67	6.74	0.90	1.05
OAory_01024400	gene162	ribose 5-phosphate isomerase A	5.3.1.6	110.63	114.25	84.93	93.38	6.80	6.85	6.43	6.56	1.03	1.10
OAory_01033640	gene1086	ribose 5-phosphate isomerase B	5.3.1.6	1834.76	2244.26	3602.78	2549.35	10.84	11.13	11.82	11.32	1.22	0.71
OAory_01077260	gene3401	transketolase tktA	2.2.1.1	312.34	471.06	335.24	374.03	8.29	8.88	8.39	8.55	1.51	1.12
OAory_01096860	gene5778	transaldolase	2.2.1.2	15.85	5.91	3.11	4.45	4.07	2.79	2.04	2.45	0.37	1.43
OAory_01097780	gene5587	transaldolase	2.2.1.2	1066.46	1240.57	916.29	825.65	10.06	10.28	9.84	9.69	1.25	0.90
OAory_01010280	gene2564	ribose-phosphate pyrophosphokinase	2.7.6.1	39.77	49.77	33.30	39.79	5.35	5.67	5.10	5.35	1.82	1.20
OAory_01057020	gene4747	ribose-phosphate pyrophosphokinase	2.7.6.1	62.95	114.88	56.33	84.56	6.00	6.86	5.84	6.42	1.82	1.50
OAory_01079480	gene2623	ribose-phosphate pyrophosphokinase	2.7.6.1	32.46	47.16	39.33	40.19	5.06	5.59	5.33	5.36	1.45	1.02
OAory_01083480	gene4023	ribokinase	2.7.1.15	89.26	115.55	58.79	58.41	6.50	6.86	5.90	5.89	1.29	0.99
OAory_01060200	gene5065	phosphoglucomutase pgmA	5.4.2.2	341.71	370.26	287.94	338.27	8.42	8.54	8.17	8.41	1.08	1.17
OAory_01091410	gene8324	phosphoglucomutase pgmA	5.4.2.2	36.46	61.26	38.66	34.08	5.23	5.96	5.31	5.13	1.68	0.88
OAory_01107650	gene6857	phosphoglucomutase pgmA	5.4.2.2	49.67	37.86	35.86	36.14	5.66	5.28	5.20	5.21	0.76	1.01

d) fatty acid biosynthesis pathway

Locus_tag	Gene_ID	Description	EC	C5_t1_fpkm	C5_t2_fpkm	C6_t1_fpkm	C6_t2_fpkm	C5_t1(log2fpkm+1)	C5_t2(log2fpkm+1)	C6_t1(log2fpkm+1)	C6_t2(log2fpkm+1)	FC_C5	FC_C6
OAory_01094140	gene8597	Acetyl-CoA carboxylase	6.4.1.2/6.3.4.14	93.96	145.35	102.86	61.19	6.57	7.19	6.70	5.96	1.55	0.59
OAory_01072760	gene10094	Fatty acid synthase alpha subunit	2.3.1.86	197.07	282.18	197.38	141.06	7.63	8.15	7.63	7.15	1.43	0.71
OAory_01072770	gene10095	Fatty acid synthase beta subunit	2.3.1.86	132.96	150.38	126.00	89.47	7.07	7.24	6.99	6.50	1.13	0.71
OAory_01027050	gene427	Acyl-CoA synthetase	6.2.1.3	0.00	0.04	0.00	0.00	0.00	0.06	0.00	0.00	-	-
OAory_01030170	gene739	Acyl-CoA synthetase	6.2.1.3	72.26	105.32	94.60	105.64	6.19	6.73	6.58	6.74	1.46	1.12
OAory_01033870	gene1109	Acyl-CoA synthetase	6.2.1.3	6.07	5.41	5.47	7.09	2.82	2.68	2.69	3.02	0.89	1.30
OAory_01048880	gene10817	Acyl-CoA synthetase	6.2.1.3	0.83	0.98	1.87	1.02	0.87	0.98	1.52	1.02	1.18	0.55
OAory_01081560	gene3831	Acyl-CoA synthetase	6.2.1.3	44.44	32.78	38.70	46.81	5.51	5.08	5.31	5.58	0.74	1.21
OAory_01092700	gene8453	Acyl-CoA synthetase	6.2.1.3	111.26	118.78	114.54	106.67	6.81	6.90	6.85	6.75	1.07	0.93
OAory_01003880	gene1924	Acyl-CoA thioesterase	3.1.2.2	28.33	26.89	27.07	27.63	4.87	4.80	4.81	4.84	0.95	1.02
OAory_01005190	gene2055	Fatty acid elongase/ELO1	2.3.1.199	35.06	99.78	64.74	48.22	5.17	6.66	6.04	5.62	2.85	0.74
OAory_01083260	gene4001	Fatty acid elongase/ELO2	2.3.1.199	44.31	70.70	62.02	70.19	5.50	6.16	5.98	6.15	1.60	1.13
OAory_01061760	gene5221	Fatty acid elongase/ELO3	2.3.1.199	1.79	0.71	1.02	1.43	1.48	0.78	1.01	1.28	0.40	1.41
OAory_01021180	gene9387	fatty acid desaturase	1.14.19.3	265.48	251.49	104.74	188.54	8.06	7.98	6.72	7.57	0.95	1.80
OAory_01045170	gene7634	fatty acid desaturase	1.14.19.3	65.60	71.65	56.06	58.03	6.06	6.18	5.83	5.88	1.09	1.04
OAory_01091420	gene8325	fatty acid desaturase	1.14.19.3	22.21	74.98	56.17	33.35	4.54	6.25	5.84	5.10	3.38	0.59
OAory_01010050	gene2541	stearoyl-CoA desaturase/ delta-9 fatty acid desaturase	1.14.19.1	974.00	1400.25	1144.82	636.67	9.93	10.45	10.16	9.32	1.44	0.56
OAory_01046260	gene7743	stearoyl-CoA desaturase/ delta-9 fatty acid desaturase	1.14.19.1	1.12	0.41	0.26	0.58	1.08	0.50	0.34	0.66	0.37	2.20
OAory_01083720	gene4047	stearoyl-CoA desaturase/ delta-9 fatty acid desaturase	1.14.19.1	398.79	1982.55	634.58	309.55	8.64	10.95	9.31	8.28	4.97	0.49
OAory_01106610	gene6753	Oleate delta-12 desaturase	1.14.19.6/1.14.19.2	640.03	1283.36	1049.53	566.55	9.32	10.33	10.04	9.15	2.01	0.54
OAory_01043420	gene7549	Oleate delta-12 desaturase	1.14.19.6/1.14.19.2	1.26	1.25	0.21	0.62	1.18	1.17	0.28	0.69	0.99	2.94

e) terpenoid backbone biosynthesis pathway

Locus_tag	Gene_ID	Description	EC	C5_t1_fpkm	C5_t2_fpkm	C6_t1_fpkm	C6_t2_fpkm	C5_t1(log2fpkm+1)	C5_t2(log2fpkm+1)	C6_t1(log2fpkm+1)	C6_t2(log2fpkm+1)	FC_C5	FC_C6
OAory_01009450	gene2481	acetyl-CoA acetyltransferase	2.3.1.9	0.00	0.05	0.00	0.00	0.00	0.07	0.00	0.00	-	-
OAory_01041580	gene7275	hydroxymethylglutaryl-CoA synthase1	2.3.3.10	82.04	69.72	50.39	117.45	6.38	6.14	5.68	6.89	0.85	2.33
OAory_01061340	gene5179	hydroxymethylglutaryl-CoA synthase2	2.3.3.10	98.72	250.09	129.49	115.35	6.64	7.97	7.03	6.86	2.53	0.89
OAory_01019760	gene9245	hydroxymethylglutaryl-coenzyme A reductase	1.1.1.34	17.00	9.71	60.52	81.70	4.17	3.42	5.94	6.37	0.57	1.35
OAory_01046250	gene7742	hydroxymethylglutaryl-coenzyme A reductase	1.1.1.34	0.00	0.14	0.00	0.16	0.00	0.18	0.00	0.21	-	-
OAory_01111690	gene10434	hydroxymethylglutaryl-coenzyme A reductase	1.1.1.34	3.72	6.16	3.29	3.46	2.24	2.84	2.10	2.16	1.66	1.05
OAory_01029170	gene639	mevalonate kinase	2.7.1.36	35.76	40.84	35.60	38.91	5.20	5.39	5.19	5.32	1.14	1.09
OAory_01041440	gene7261	phosphomevalonate kinase	2.7.4.2	53.42	182.46	123.85	56.04	5.77	7.52	6.96	5.83	3.42	0.45
OAory_01029820	gene704	diphosphomevalonate decarboxylase	4.1.1.33	69.45	69.93	63.67	60.94	6.14	6.15	6.02	5.95	1.01	0.96
OAory_01026810	gene403	isopentenyl-diphosphate delta-isomerase	5.3.3.2	330.75	203.32	365.14	406.69	8.37	7.67	8.52	8.67	0.61	1.11
OAory_01083570	gene4032	farnesyl diphosphate synthase	2.5.1.1/2.5.1.10	284.80	138.13	167.67	184.76	8.16	7.12	7.40	7.54	0.48	1.10
OAory_01023330	gene55	geranylgeranyl diphosphate synthase, type III	2.5.1.1/2.5.1.10/2.5.1.29	0.00	0.24	0.00	7.37	0.00	0.31	0.00	3.06	-	-

f) steroid biosynthesis pathway

Locus_tag	Gene_ID	Description	EC	C5_t1_fpkm	C5_t2_fpkm	C6_t1_fpkm	C6_t2_fpkm	C5_t1(log2fpkm+1)	C5_t2(log2fpkm+1)	C6_t1(log2fpkm+1)	C6_t2(log2fpkm+1)	FC_C5	FC_C6
OAory_01020110	gene9280	squalene monooxygenase	1.14.14.17 (1.14.13.132)	121.19	447.82	170.45	81.35	6.93	8.81	7.42	6.36	3.70	0.48
OAory_01081770	gene3852	lanosterol synthase	5.4.99.7	25.59	30.36	19.36	25.08	4.73	4.97	4.35	4.70	1.19	1.30
OAory_01031230	gene845	sterol 14alpha-demethylase	1.14.14.154 (1.14.13.70)	131.72	243.80	115.33	105.07	7.05	7.94	6.86	6.73	1.85	0.91
OAory_01064820	gene5527	sterol 14alpha-demethylase	1.14.14.154 (1.14.13.70)	743.76	1387.36	385.14	180.96	9.54	10.44	8.59	7.51	1.87	0.47
OAory_01028620	gene584	Delta14-sterol reductase	1.3.1.70	90.66	166.27	50.95	34.58	6.52	7.39	5.70	5.15	1.83	0.68
OAory_01050620	gene10991	Delta14-sterol reductase	1.3.1.70	33.04	64.03	18.33	13.87	5.09	6.02	4.27	3.89	1.94	0.76
OAory_01043030	gene7420	methylsterol monooxygenase	1.14.18.9 (1.14.13.72)	278.48	1812.49	542.23	106.76	8.13	10.82	9.09	6.75	6.51	0.20
OAory_01021800	gene11355	methylsterol monooxygenase	1.14.18.9 (1.14.13.72)	922.59	2030.35	833.17	421.01	9.85	10.99	9.70	8.72	2.20	0.51
OAory_01070590	gene9877	sterol-4alpha-carboxylate 3-dehydrogenase	1.1.1.170	29.51	14.18	20.36	16.54	4.93	3.92	4.42	4.13	0.48	0.81
OAory_01057530	gene4798	3-keto steroid reductase	1.1.1.270	18.07	22.94	13.04	17.02	4.25	4.58	3.81	4.17	1.27	1.31
OAory_01037400	gene1462	cholestenol Delta-isomerase	5.3.3.5	37.49	55.41	19.36	11.36	5.27	5.82	4.35	3.63	1.48	0.59
OAory_01097390	gene5831	cholestenol Delta-isomerase	5.3.3.5	61.81	38.65	26.92	15.51	5.97	5.31	4.80	4.05	0.63	0.58
OAory_01014460	gene8715	lathosterol oxidase/ C-5 sterol desaturase	1.14.19.20	133.46	245.13	118.19	86.69	7.07	7.94	6.90	6.45	1.84	0.73
OAory_01107080	gene6800	7-dehydrocholesterol reductase	1.3.1.21	0.37	1.81	1.87	2.29	0.45	1.49	1.52	1.72	0.95	1.22
OAory_01028520	gene574	TAG lipase (RefSeq - hypothetical protein)	3.1.1.13	7.47	7.10	8.59	10.37	3.08	3.02	3.26	3.51	1.58	1.21
OAory_01049650	gene10894	sterol O-acyltransferase	2.3.1.26	21.95	34.68	18.16	22.00	4.52	5.16	4.26	4.52	1.58	1.21
OAory_01027770	gene499	sterol 24-C-methyltransferase (ERG6)	2.1.1.41	45.24	154.68	216.86	40.58	5.53	7.28	7.77	5.38	3.42	0.19
OAory_01089740	gene8157	sterol 24-C-methyltransferase (ERG6)	2.1.1.41	64.39	94.99	53.37	66.73	6.03	6.58	5.76	6.08	1.48	1.25
OAory_01058890	gene4934	C-8 sterol isomerase (ERG2)	5.-.-.-	132.24	117.87	71.86	95.63	7.06	6.89	6.19	6.59	0.89	1.33
OAory_01064390	gene5484	sterol 22-sedaturase (cytochrome P450)	1.14.19.41	139.20	376.04	157.00	158.02	7.13	8.56	7.30	7.31	2.70	1.01
OAory_01036990	gene1421	ERG4/ERG24 ergosterol biosynthesis protein	1.3.1.71	8.02	3.07	6.78	7.85	3.17	2.02	2.96	3.15	0.38	1.16
OAory_01080910	gene3766	ERG4/ERG24 ergosterol biosynthesis protein	1.3.1.71	85.94	78.12	75.77	89.34	6.44	6.31	6.26	6.50	0.91	1.18

g) amino acid metabolism

Locus_tag	Gene_ID	Description	EC	C5_t1_fpkm	C5_t2_fpkm	C6_t1_fpkm	C6_t2_fpkm	C5_t1(log2fpkm+1)	C5_t2(log2fpkm+1)	C6_t1(log2fpkm+1)	C6_t2(log2fpkm+1)	FC_C5	FC_C6
OAory_01081090	gene3784	glutamate synthase (NADPH/NADH)	1.4.1.13/1.4.1.14	61.09892127	120.925349	95.86545484	114.6156323	5.96	6.93	6.60	6.85	1.98	0.84
OAory_01030390	gene761	glutamate dehydrogenase (NADP+)	1.4.1.4	165.9510707	601.327891	190.5518625	205.8660216	7.38	9.23	7.58	7.69	3.62	0.93
OAory_01046690	gene7786	glutamate decarboxylase	4.1.1.15	238.933623	554.0039513	295.9495699	248.1599511	7.91	9.12	8.21	7.96	2.32	1.19
OAory_01034360	gene1158	adenylosuccinate lyase	4.3.2.2	40.75252944	118.4937495	59.7723524	69.13192567	5.38	6.90	5.93	6.13	2.91	0.86
OAory_01015680	gene8837	catalase	1.11.1.6	7.27939043	34.00239976	55.07977954	19.13142863	3.05	5.13	5.81	4.33	4.67	2.88
OAory_01071500	gene9968	catalase	1.11.1.6	207.6731881	413.7913849	268.0881527	186.7271324	7.71	8.70	8.07	7.55	1.99	1.44
OAory_01098850	gene5977	catalase	1.11.1.6	78.05124183	30.42148735	108.811829	21.00769175	6.30	4.97	6.78	4.46	0.39	5.18
OAory_01089930	gene8176	glutamine synthetase	6.3.1.2	26.44845189	8.643281404	20.23976468	17.09623734	4.78	3.27	4.41	4.18	0.33	1.18
OAory_01105320	gene6624	tyrosinase	1.14.18.1	14.73676107	43.97705359	7.536447974	10.27911605	3.98	5.49	3.09	3.50	2.98	0.73
OAory_01026170	gene339	tyrosinase	1.14.18.1	0.343714894	0.26072974	1.392318367	11.75926409	0.43	0.33	1.26	3.67	0.76	0.12
OAory_01064800	gene5525	4-hydroxyphenylpyruvate dioxygenase	1.13.11.27	26.27148426	220.2295024	10.20847361	2.836814077	4.77	7.79	3.49	1.94	8.38	3.60
OAory_01018570	gene9126	primary-amine oxidase	1.4.3.21	17.89516814	37.61783078	12.36829	8.211246997	4.24	5.27	3.74	3.20	2.10	1.51
OAory_01012920	gene2828	primary-amine oxidase	1.4.3.21	12.86589936	129.5181298	16.56089843	35.92971405	3.79	7.03	4.13	5.21	10.07	0.46
OAory_01048490	gene10778	alcohol dehydrogenase, propanol-preferring	1.1.1.1	385.0818276	3173.085018	1269.673894	437.7604185	8.59	11.63	10.31	8.78	8.24	2.90
OAory_01034810	gene1203	hydroxymethylglutaryl-CoA lyase	4.1.3.4	41.14618408	91.62236189	25.56127275	29.13795998	5.40	6.53	4.73	4.91	2.23	0.88
OAory_01102790	gene6371	hydroxymethylglutaryl-CoA lyase	4.1.3.4	57.27297792	16.940178	100.1303712	45.09979354	5.86	4.17	6.66	5.53	0.30	2.22
OAory_01061340	gene5179	hydroxymethylglutaryl-CoA synthase	2.3.3.10	98.72179819	250.0858205	129.4883263	115.3547985	6.64	7.97	7.03	6.86	2.53	1.12
OAory_01041810	gene7298	3-hydroxyisobutyrate dehydrogenase	1.1.1.31	33.44854916	326.090171	3.652738208	3.23766824	5.11	8.35	2.22	2.08	9.75	1.13
OAory_01017030	gene8972	aldehyde dehydrogenase (NAD+)	1.2.1.3	82.48999376	376.0584077	97.07279137	73.08149626	6.38	8.56	6.62	6.21	4.56	1.33
OAory_01026950	gene417	3-hydroxyisobutyrate dehydrogenase	1.1.1.31	127.9892823	87.92537441	131.180743	63.63542897	7.01	6.47	7.05	6.01	0.69	2.06
OAory_01060580	gene5103	methylglutaconyl-CoA hydratase	4.2.1.18	66.71441339	24.14054161	33.57017284	33.59462	6.08	4.65	5.11	5.11	0.36	1.00
OAory_01026520	gene374	aldehyde dehydrogenase	1.2.1.3	549.1035239	123.2037897	549.103524	749.5597629	9.10	6.96	9.10	9.55	0.22	0.73
OAory_01096340	gene5726	cysteine dioxygenase	1.13.11.20	30.1528528	85.78143632	165.4959115	91.4494298	4.96	6.44	7.38	6.53	2.84	1.81
OAory_01029590	gene681	5-methyltetrahydropteroyltriglutamate--homocysteine met	2.1.1.14	263.0724737	606.0819239	397.552406	482.8001331	8.04	9.25	8.64	8.92	2.30	0.82
OAory_01081590	gene3834	O-acetylhomoserine/O-acetylserine sulfhydrylase	2.5.1.49/2.5.1.47	50.88894961	151.9531129	50.87688852	29.11312979	5.70	7.26	5.70	4.91	2.99	1.75
OAory_01004000	gene1936	homoserine O-acetyltransferase	2.3.1.31	51.31769165	16.58598868	62.11342492	46.78980014	5.71	4.14	5.98	5.58	0.32	1.33
OAory_01028840	gene606	threonine dehydratase	4.3.1.19	44.70160884	108.8368406	40.55543958	52.48792772	5.51	6.78	5.38	5.74	2.43	0.77
OAory_01003010	gene1837	phosphoglycerate mutase	5.4.2.11	242.2178743	666.3779232	360.8432227	294.3004032	7.93	9.38	8.50	8.21	2.75	1.23
OAory_01070940	gene9912	phosphoglycerate mutase	5.4.2.11	233.9110791	515.6978955	330.3725895	180.142126	7.88	9.01	8.37	7.50	2.20	1.83
OAory_01035660	gene1288	tryptophan synthase	4.2.1.20	27.58717812	57.98868625	39.66444779	40.31277598	4.84	5.88	5.35	5.37	2.10	0.98
OAory_01001620	gene1698	choline dehydrogenase	1.1.99.1	1.851673241	0.860325238	1.006350302	22.55808396	1.51	0.90	1.00	4.56	0.46	0.04
OAory_01076010	gene3276	glutathione S-transferase	2.5.1.18	87.98021881	61.08980217	196.5651465	92.3493031	6.48	5.96	7.63	6.54	0.69	2.13
OAory_01010430	gene2579	glutathione S-transferase	2.5.1.18	173.5302221	253.0656884	207.8304677	38.33992389	7.45	7.99	7.71	5.30	1.46	5.42
OAory_01061170	gene5162	glutathione S-transferase	2.5.1.18	328.2976082	259.7411828	643.0818993	161.8594566	8.36	8.03	9.33	7.35	0.79	3.97
OAory_01102470	gene6339	indoleamine 2,3-dioxygenase	1.13.11.52	367.0683785	340.1169718	731.4748634	159.2225986	8.52	8.41	9.52	7.32	0.93	4.59
OAory_01056150	gene4660	kynureninase	3.7.1.3	7.539368659	25.07855385	6.818941358	4.286090465	3.09	4.70	2.97	2.40	3.33	1.59
OAory_01112050	gene10470	acetamidase	3.5.1.4	11.57236427	0.566347017	10.08113298	16.14022515	3.65	0.65	3.47	4.10	0.05	0.62
OAory_01035620	gene1284	aminotransferase family protein (LolT)	4.4.1.36	47.33179405	19.68913986	74.1054921	35.56839858	5.59	4.37	6.23	5.19	0.42	2.08
OAory_01051860	gene11115	gamma-glutamyltranspeptidase / glutathione hydrolase	2.3.2.2/3.4.19.13	47.87368467	18.42447207	31.60210981	35.29660349	5.61	4.28	5.03	5.18	0.38	0.90
